# Causal association of smoking, blood lipids, and bladder cancer: Insights from a multivariable and mediation mendelian randomization investigation

**DOI:** 10.7150/jca.92306

**Published:** 2024-02-12

**Authors:** Houyi Wei, Xiangqun Cheng, Gang Wang, Zhilong Li, Wenzhi Du, Lingao Ju, Danni Shan, Mengxue Yu, Yayun Fang, Kaiyu Qian, Yi Zhang, Yu Xiao, Xinghuan Wang

**Affiliations:** 1 Department of Urology, Zhongnan Hospital of Wuhan University, Wuhan, China.; 2 Physical Examination Center, Zhongnan Hospital of Wuhan University, Wuhan, China.; 3 Department of Biological Repositories, Human Genetic Resource Preservation Center of Hubei Province, Hubei Key Laboratory of Urological Diseases, Zhongnan Hospital of Wuhan University, Wuhan, China.; 4 Euler Technology, ZGC Life Sciences Park, Beijing, China.; 5 Center for Quantitative Biology, School of Life Sciences, Peking University, Beijing, China.; 6 Medical Research Institute, Frontier Science Center of Immunology and Metabolism, Wuhan University, Wuhan, China.

**Keywords:** smoking, blood lipids, bladder cancer, Mendelian randomization, causal association

## Abstract

We used Mendelian randomization (MR) to examine the relationship between smoking, various categories of blood lipids, and bladder cancer (BLCA). Data for this study were drawn from the genome-wide association studies of the GSCAN consortium (~1.2 million participants), a subset of the UK Biobank (~120,000 participants), and the FinnGen consortium (2,072 cases and 307,082 controls). Initially, we utilized inverse variance weighted (IVW), complementary and sensitivity analyses, multivariable MR, and meta-analysis to confirm the association between blood lipids and BLCA. We then performed mediation MR to elucidate the relationship between smoking, blood lipids, and BLCA. Our analysis identified five lipids, including triglycerides in very large HDL, cholesterol in small VLDL, free cholesterol in very large HDL, total free cholesterol, and apolipoprotein B, as having strong and inverse associations with BLCA. These lipids demonstrated no heterogeneity or pleiotropy and exhibited consistent direction and magnitude across IVW, weighted median, and MR-Egger analyses. Our mediation MR further revealed that triglycerides in very large HDL and cholesterol in small VLDL could reduce the impact of smoking on BLCA, mediating -4.3% and -4.5% of the effect, respectively. In conclusion, our study identified five lipids exhibiting a robust inverse relationship with BLCA, two of which can buffer the impact of smoking on BLCA.

## Introduction

Bladder cancer (BLCA) is the 10^th^ most frequently diagnosed cancer worldwide 1. Smoking and occupational exposure are the main risk factors for BLCA. Besides, BLCA could be influenced by alcohol consumption, BMI, gender, diet, medical usage, etc. Although some progress has been made, the etiology of BLCA still remains partly understood and needs further exploration 2.

During the past several years, whether blood lipids level can affect cancer risk has attracted much attention. Many studies have indicated that some specific lipids, such as polyunsaturated fatty acids 3, 4, cholesterol 5-7, HDL cholesterol 8-10, LDL cholesterol 11, apolipoprotein A1 12, 13, are inversely associated with cancer risk. However, other studies have reported a positive association 14-17 or no association 18, 19 between lipids and cancer. Thus, although many studies have been carried out on this topic, the associations between lipids and cancer are controversial. Smoking is thought to be associated with a wide range of diseases, including dyslipidemia 20-24 and several other cancers 25-27. However, the associations between smoking, blood lipids and BLCA have rarely been explored. Therefore, more robust evidence is urgently needed to clarify this topic.

Mendelian randomization (MR) is a newly developed and multifaceted analytic approach in epidemiology research 28. Single nucleotide polymorphisms (SNPs) are used as instrumental variables (IVs) to proxy for the exposures of interest, thus mimicking the process of randomized controlled trials (RCTs) since SNPs are randomly allocated during meiosis 29. As genotype formation is fixed at conception and before disease onset, the result of MR is less likely to be biased by reverse causation. In addition, the effect of confounding factors is also eliminated in MR because of the assumption that IVs influence the outcome only through the exposure of interest 30, 31.

Thus, the associations between smoking, blood lipids and BLCA have rarely been explored. Conventional observational studies might be affected by confounding factors and reverse causation, and it is difficult to carry out RCTs on this topic. Therefore, we chose MR to explore their association.

## Materials and methods

### Study design

**Figure 1:**
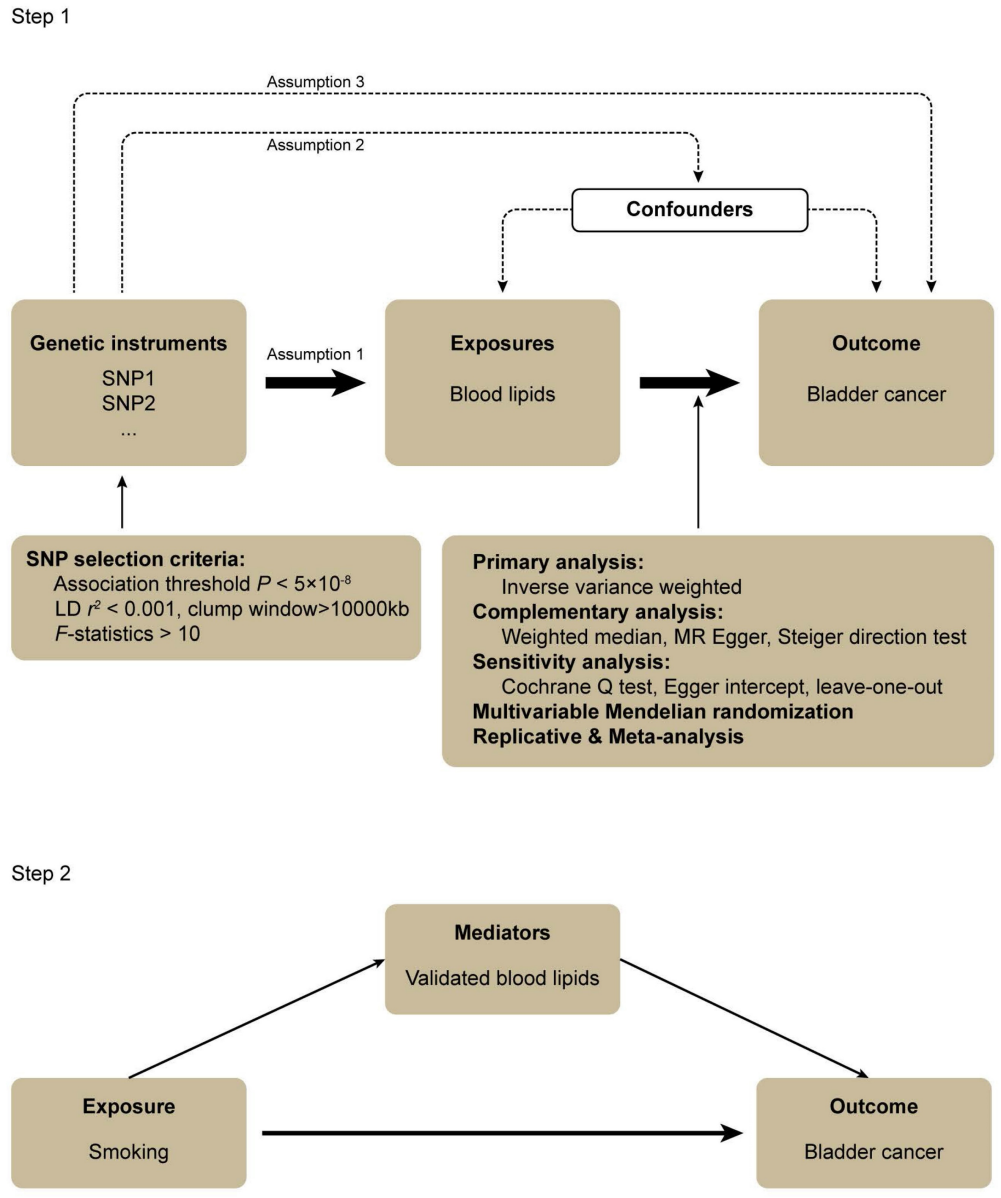
Flowchart of this mendelian randomization study.

Three assumptions should be followed for MR design: (1) the IVs should have a robust association with the exposure, (2) the IVs should have no association with confounding factors, and (3) the IVs should influence the outcome only through the exposure of interest (Figure 1) 28.

In step 1, the associations between a wide range of subdivided blood lipids and BLCA were systematically evaluated. The genome-wide association study (GWAS) data for exposures and outcomes were derived from the UK Biobank (UKB) cohort study and FinnGen consortium, respectively. The random effect inverse-variance weighted (IVW) was performed for the primary analysis. Complementary and sensitivity analyses were also conducted to examine the results of the primary analysis. After this, a multivariable MR analysis was performed to attenuate the influence of confounders that might cause pleiotropy. We subsequently carried out the replicative and meta-analysis to draw a robust conclusion. In step 2, a mediation MR was conducted to explore the association between smoking, blood lipids validated in step 1 and BLCA. All the GWAS data were obtained from European populations hence preventing demographic stratification bias (Figure 1).

All the statistical analyses were performed using the MendelianRandomization (Version 0.7.0), TwoSampleMR package (Version 0.5.6), MVMR (Version 0.3), RadialMR (version 1.0) in the R program (Version 4.2.1), the Reviewer Manager software (Version 5.4.1).

### GWAS data for blood lipids and smoking

The UKB cohort study is a large-scale biomedical database and research resource that contains nearly 500,000 adults (aged 37~73 at baseline) who were recruited between 2006 and 2010. High-throughput detection of blood lipids and metabolites was performed by Nightingale Health Ltd in non-fasting (average 4 hours after last meal) plasma samples from a randomly selected UKB subset of approximately 120,000 participants 32, 33. This metabolomic profiling was generated by using Nightingale Health Ltd's nuclear magnetic resonance-based metabolomics platform, and comprised a panel of 249 metabolic measures (168 measures in absolute levels and 81 ratio measures), which included lipoprotein lipids, fatty acids and small molecules, etc. We selected 230 biomarkers of lipid metabolism from the 249 metabolic measures. These data could be obtained from the website: *https://gwas.mrcieu.ac.uk/*. Then, we conducted the replicative analysis and meta-analysis to validate our results by using GWAS data from the study of Kettunen *et al*. 34, which detected 123 circulating metabolites for 24,925 individuals. For mediation MR, the summary-level GWAS data for cigarettes smoked per day was obtained from the GWAS and Sequencing Consortium of Alcohol and Nicotine use, which included nearly 1.2 million individuals 35.

### GWAS data for BLCA

GWAS data for BLCA was obtained from the seventh release of the FinnGen consortium (*https://www.finngen.fi/fi*), which comprised 309,154 Finnish men and women, and individuals with excess heterozygosity (±4 SD), a genotype missingness over 5%, ambiguous sex and non-Finnish ancestry were omitted. The GWAS analyses were adjusted for age, sex and the first ten genetic principal components. The definition of disease followed the criteria of the International Classification of Diseases codes (8^th^, 9^th^ and 10^th^ revisions) with information obtained from registries all over the country 36.

### Instrument selection

IVs should have a robust association with exposures by reaching genome-wide significance (*P* < 5×10^-8^). Besides this, the linkage disequilibrium of the included IVs was avoided (*r^2^* < 0.001 and clump window >10000 kb). *F* statistic for each SNP was calculated. SNPs with *F* < 10 were omitted to ensure that every SNP had sufficient statistical strength.

### Primary and complementary analysis

For the primary analysis, a random effect IVW approach was used to validate whether there were significant causal associations (*P*<0.05) between blood lipids and BLCA. By combining the Wald ratios from each SNP, IVW can ultimately generate a pooled estimate and is usually used in MR studies for primary analysis 37. The IVW can provide unbiased estimates since it assumes that all the IVs are valid. However, IVW could also be easily influenced by pleiotropy bias.

In the complementary analysis, the weighted median and MR-Egger were calculated to validate the results that were significant in IVW analysis. Weighted median is based on the assumption that at least half of the IVs are valid 38. MR-Egger regression can adjust for pleiotropy and provide consistent estimates even if all the IVs are invalid, but can reduce statistical power at the same time 39. Then, a Steiger test was performed to validate whether the causal inference direction was biased by reverse causation, with *P* > 0.05 suggesting that the direction could be biased 40.

The Cochran Q test was used to detect heterogeneity (*P* < 0.05 suggested the existence of heterogeneity). And *I^2^* of 25%, 50% and 75% represented low, medium and high heterogeneity, respectively 41. We performed MR radial analysis for heterogeneous results to identify and exclude outliers. Egger intercept was used to examine whether horizontal pleiotropy existed 39. The leave-one-out method was used to exclude each SNP one by one and calculate the combined effect of remaining SNPs, thus clarifying whether the causal association between blood lipids and BLCA was caused mainly by the effect of a single SNP.

### Multivariable MR and meta-analysis

To accomplish the second MR assumption, we explored the PhenoScanner database to explore whether there were genome-wide significant associations (*P* < 5×10^-8^) between included SNPs and potential confounders. After exploration, we performed multivariable MR analysis by acquiring SNPs from large scale GWAS studies for smoking 35, alcohol consumption 35 and coronary artery disease 42, and UKB data for body mass index, hence adjusting for indirect pleiotropic pathways. To validate the robustness of our results, the replicative and meta-analysis were conducted, with GWAS data for blood lipids obtained from the study of Kettunen *et al*. 34.

### Mediation MR

In step 2 (Figure 1), we performed a mediation MR using the blood lipids that were validated through the abovementioned analyses. Smoking was regarded as the exposure factor, blood lipids were used as the mediators and BLCA was the outcome. We performed two-sample MR and used IVW as our main approach to estimate the effect of smoking on BLCA (β1) and blood lipids (β2), respectively. A multivariable MR was subsequently conducted to estimate the effect of each blood lipid on BLCA (β3) after adjusting for the genetic effect of smoking. Thus, the proportion of the total effect mediated by each blood lipid separately was estimated as the indirect effect divided by the total effect: β2×β3/β1.

## Results

### Genetic instruments selection

A total of 230 blood lipids were selected for MR analysis. The number of SNPs for the exposures of interest ranged from 16 to 86. The minimum *F* statistic was 19.4 for all the SNPs (Supplementary Table S1).

### Primary MR analysis

**Figure 2:**
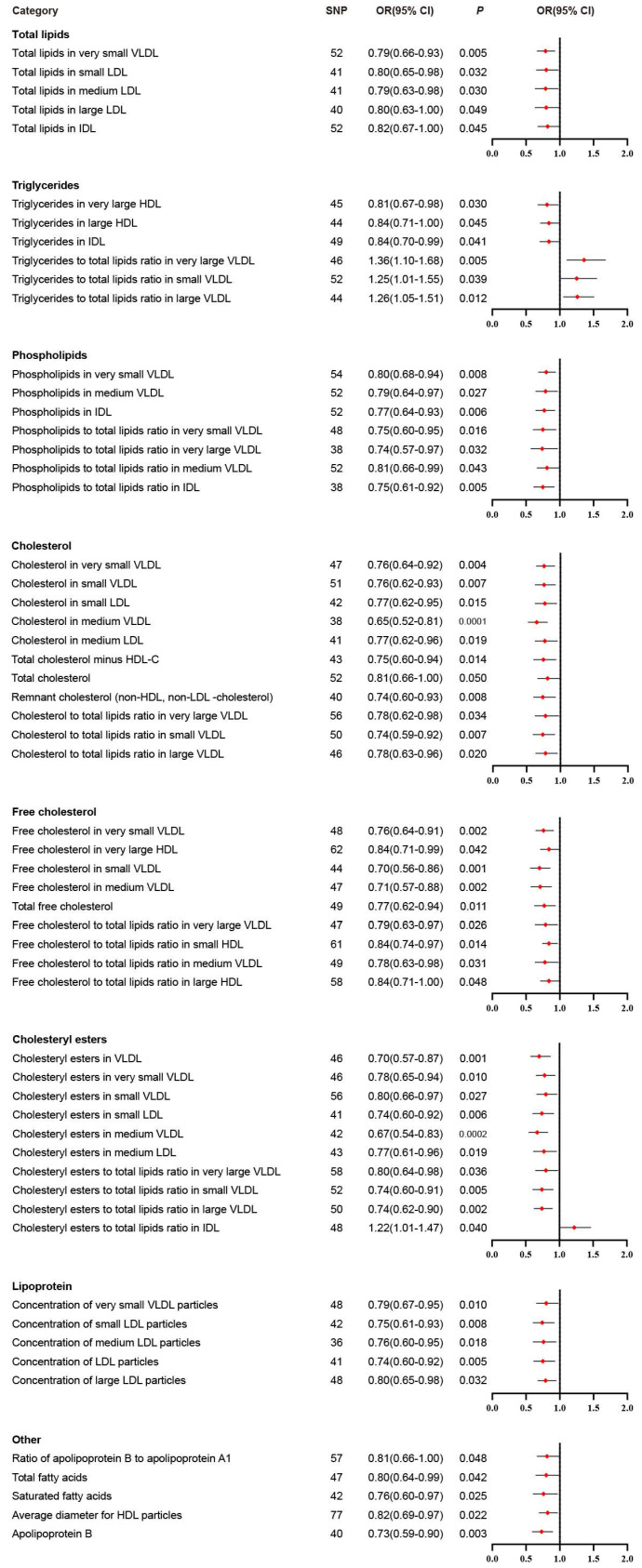
Significant results in primary inverse variance weighted analysis.

We performed IVW analysis for all 230 blood lipids, and 58 of them were significantly associated with BLCA (Figure 2). According to their characteristics, the 58 blood lipids were assigned to 8 groups including total lipids, triglycerides, phospholipids, cholesterol, free cholesterol, cholesteryl esters, lipoprotein and other. The most significant lipids were cholesterol in medium VLDL (OR 0.65, 95% CI 0.52-0.81, *P* = 0.0001), free cholesterol in very small VLDL (OR 0.76, 95% CI 0.64-0.91, *P* = 0.002), free cholesterol in small VLDL (OR 0.70, 95% CI 0.56-0.86, *P* = 0.001), free cholesterol in medium VLDL (OR 0.71, 95% CI 0.57-0.88, *P* = 0.002), cholesteryl esters in VLDL (OR 0.70, 95% CI 0.57-0.87, *P* = 0.001), cholesteryl esters in medium VLDL (OR 0.67, 95% CI 0.54-0.83, *P* = 0.0002), cholesteryl esters to total lipids ratio in large VLDL (OR 0.74, 95% CI 0.62-0.90, *P* = 0.002), and apolipoprotein B (OR 0.73, 95% CI 0.59-0.90, *P* = 0.003).

### Complementary analysis

Based on the significant results of IVW, we performed weighted median and MR-Egger for further validation (Supplementary Table S2). Apart from four results (i.e., total lipids in large LDL, total cholesterol minus HDL-C, cholesterol to total lipids ratio in very large VLDL, and cholesteryl esters to total lipids ratio in very large VLDL. These four results were not used for further analysis), consistent directions and magnitudes were shown in the scatter plot results of IVW, weighted median and MR-Egger analysis (Supplementary Figure S1). All Steiger *P* < 0.05 proved that the directions of these causal associations were true and free from reverse causation (Supplementary Table S2).

By performing sensitivity analysis, Cochran Q-derived *P* and *I^2^* showed all the 58 results had no heterogeneity (Supplementary Table S2). Additionally, MR-Egger intercept showed that in addition to two other results (i.e., phospholipids in medium VLDL, total cholesterol minus HDL-C. These two results were omitted from the subsequent analysis), horizontal pleiotropy was not detected (Supplementary Table S2). The results of leave-one-out analysis were presented in Supplementary Figure S2.

### Multivariable MR and meta-analysis

After exploring PhenoScanner database, we found associations between the included IVs and other confounders, such as smoking, alcohol consumption, body mass index and coronary artery disease. Then the multivariable MR analysis was conducted to adjust for these confounders, thus preventing correlation of pleiotropic pathways. After conducting multivariable MR, 23 blood lipids remained significant (Supplementary Figure S3).

**Figure 3:**
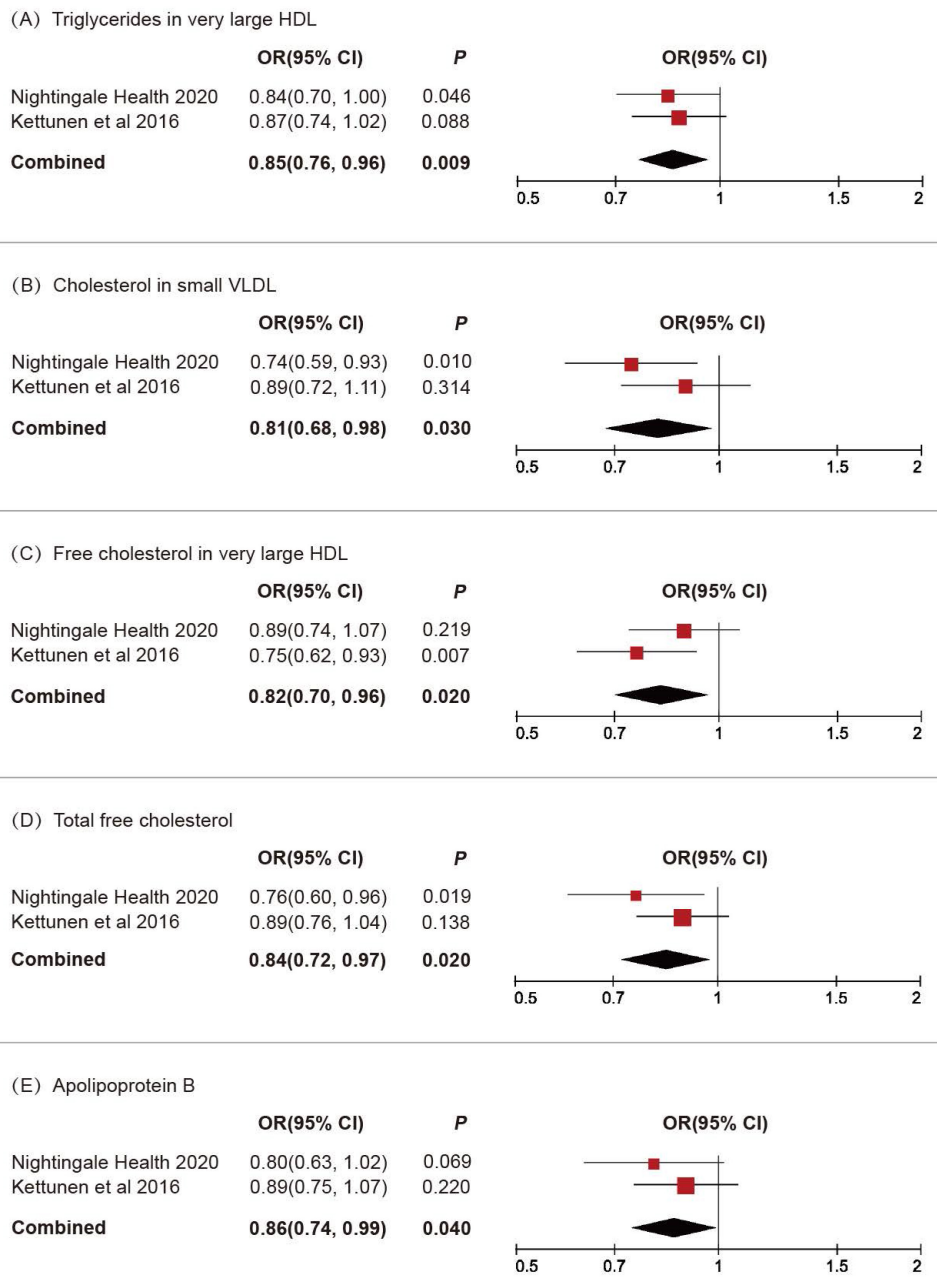
Replicative and meta-analysis results for the causal associations between blood lipids and BLCA.

We subsequently conducted a replicative analysis using additional GWAS data for blood lipids and integrated the results. Through multivariable MR and meta-analysis, five lipids were confirmed to be associated with BLCA (Figure 3): triglycerides in very large HDL (OR 0.85, 95% CI 0.76-0.96, *P* = 0.009), cholesterol in small VLDL (OR 0.81, 95% CI 0.68-0.98, *P* = 0.03), free cholesterol in very large HDL (OR 0.82, 95% CI 0.70-0.96, *P* = 0.02), total free cholesterol (OR 0.84, 95% CI 0.72-0.97, *P* = 0.02), apolipoprotein B (OR 0.86, 95% CI 0.74-0.99, *P* = 0.04).

### Mediation MR

**Figure 4:**
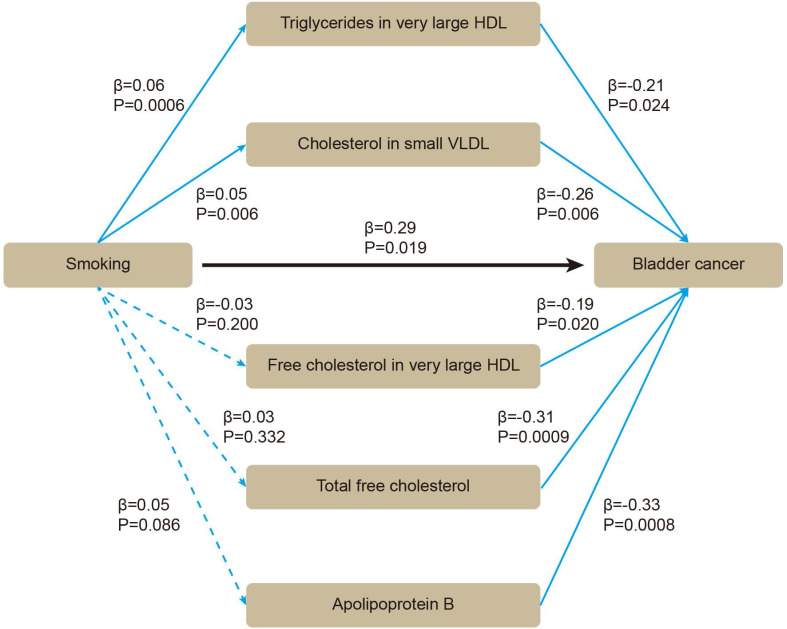
The association between smoking, blood lipids and BLCA incidence were determined by mediation mendelian randomization.

Based on these five lipids, we performed mediation MR. By using two-sample MR, we evaluated whether smoking affected BLCA or blood lipids. All the SNPs were sufficient (Supplementary Table S3). Smoking was positively associated with BLCA (β = 0.29 [95% CI 0.05, 0.53], *P* = 0.019), without heterogeneity or pleiotropy (Supplementary Table S4). For mediators, the SNPs were sufficient (Supplementary Table S5). Smoking was positively associated with triglycerides in very large HDL (β = 0.06 [95% CI 0.03, 0.09], *P* = 0.0006), and with cholesterol in small VLDL (β = 0.05 [95% CI 0.01, 0.08], *P* = 0.006) (Supplementary Table S6). After adjusting for smoking in multivariable MR, triglycerides in very large HDL (β = -0.21 [95% CI -0.40, -0.03], *P* = 0.024), cholesterol in small VLDL (β = -0.26 [95% CI -0.45, -0.08], *P* = 0.006) still showed inverse association with BLCA (Figure 4). Therefore, an indirect effect of smoking on BLCA was observed through triglycerides in very large HDL and cholesterol in small VLDL, with a mediated proportion of -4.3% and -4.5%, respectively.

## Discussion

The purpose of our research was to assess the associations between smoking, a wide range of subdivided blood lipids and BLCA risk, which, to the best of our knowledge, has not been examined before. Through two-sample MR, we identified 58 significant results from a total of 230 blood lipids. Most of the results had no heterogeneity or pleiotropy, and showed consistent direction and magnitude in IVW, weighted median and MR-Egger. Through multivariable MR and meta-analysis, we validated the inverse causal association between five lipids and BLCA, including triglycerides in very large HDL, cholesterol in small VLDL, free cholesterol in very large HDL, total free cholesterol, and apolipoprotein B. Through mediation MR, we observed that triglycerides in very large HDL and cholesterol in small VLDL could attenuate the effect of smoking on BLCA with a mediated proportion of -4.3% and -4.5%, respectively.

Previous observational studies have explored the association between lipids and cancer risk. There was an inverse association between total cholesterol and all-cancer incidence 6, 12. Results from the Women's Health Study and the Supplementation en Vitamines et Mineraux Antioxydants Study indicated that both HDL cholesterol and apolipoprotein A1 had inverse associations with total cancer risk 12, 43. Since type 2 diabetes was always accompanied by metabolic disorders, some studies chose patients with type 2 diabetes to study and also found that HDL cholesterol was inversely associated with cancer risk 10, 44. One study combined the results of 24 RCTs and revealed that HDL cholesterol was inversely associated with cancer risk, which remained consistent after adjusting for LDL cholesterol, age, BMI, diabetes, sex, and smoking 8. Another study integrated 15 RCTs and reported that on-treatment (statins) LDL cholesterol was associated with a reduced incidence of cancer 45. These two studies summarized the results from RCTs and could provide higher level evidence. However, the patients included in these RCTs were receiving lipid-lowering drugs and the association between lipids and cancer risk could be biased by drug usage. In addition, they only reported the overall cancer incidence, without specific incidence for different types of cancer, for which the effect could be totally different.

There were also studies focusing on specific types of cancer. Total cholesterol was inversely associated with liver and colorectal cancer risk 5, 7. HDL cholesterol could decrease colon cancer risk 9. Triglycerides could increase the risk of cancer in caecum and transverse colon, lung, rectal, thyroid, prostate, and gynaecological 15, 19. n-3 polyunsaturated fatty acid decreased the risk of oral and pharyngeal, oesophageal, colon and ovarian cancer 3. Apolipoprotein A presented to be inversely associated with hepatic flexure cancer and lung cancer risk 13, 19. Several studies have showed that both higher and lower levels of some lipids could increase biliary tract cancer and lung cancer risk, which might indicate that dyslipidemia per se was a modifiable risk factor for some specific cancers 46, 47. For BLCA, several studies have indicated that omega-3 fatty acids, triglyceride, and total cholesterol were not associated with BLCA risk 6, 15, 18. However, another study showed that triglyceride and total cholesterol might be risk factors for BLCA 17.

These observational studies provided controversial and confusing results, probably due to reverse causation, sample size and selection bias. Confounding factors such as diet, body mass index, and drug usage may also influence lipids and cancer, however, it is difficult to conduct RCTs to clarify this association. By utilizing genetically predicted lipids, MR approach has the potential to mimic RCTs, thus overcoming limitations inherent in traditional observational studies and providing evidence to clarify this causal association more rigorously.

Several MR studies have been conducted to explore the causal association between lipids and cancer risk. One MR study indicated that there was a causal and negative association between serum triglycerides and overall cancer incidence, but this association was not significant for LDL cholesterol 48. Two MR studies provided strong evidence that HDL, LDL and HDL cholesterol might increase breast cancer risk, whereas LDL cholesterol could not 49, 50. Lipoprotein A presented to increase the risk of prostate cancer 51. Additionally, HDL cholesterol was reported to increase the non-endometrioid endometrial cancer risk, whereas LDL cholesterol was inversely associated with all histology types of endometrial cancer 52. Currently, only one MR study has concluded that HDL cholesterol, LDL cholesterol, and triglycerides were not causally related to BLCA; however, this study evaluated only three exposures and had a relatively small sample size of only 400 BLCA cases. 48. Therefore, the causal association between lipids and BLCA needs to be further explored using MR.

Lipids refer to a group of complex compounds. Different lipids could have completely different biological functions. For instance, LDL cholesterol and HDL cholesterol, which play opposite roles in intracellular cholesterol homeostasis, can affect cardiovascular 53 and malignant diseases differently 49, 50, 52. Monounsaturated fatty acid and polyunsaturated fatty acid were associated with different susceptibilities to peroxidation, and thus play different roles in several cellular biological processes, such as stress response, apoptosis and ferroptosis 54. Thus, in this study, we focused on the risk factor for BLCA and preformed a systematic screening of these subdivided blood lipids to determine which types of blood lipids play the leading role in BLCA formation.

We discovered that five lipids, including triglycerides in very large HDL, cholesterol in small VLDL, free cholesterol in very large HDL, total free cholesterol, and apolipoprotein B, could reduce BLCA risk. The effects of the former three blood lipids on cancer risk have not been reported. Apolipoprotein B could reduce breast cancer risk in women and increase lung and colorectal cancer risk in both genders 13. In addition, both low and high levels of apolipoprotein B were associated with an increased risk of biliary tract cancer 46. According to previous reports, free cholesterol was found to induce endoplasmic reticulum stress thus promoting apoptosis of lymphoma cells 55. Moreover, the accumulation of free cholesterol within the cells could decrease AKT phosphorylation and inhibit the invasion of lung cancer cells 56. The above studies also support our finding of an inverse association between total free cholesterol and BLCA risk.

Smoking is one of the most important risk factors for BLCA 25. One MR study indicated that smoking initiation and lifetime smoking were both associated with multiple diseases, including several cancers for the bladder, lung, head and neck, etc. 26. Another MR study explored the association between smoking and BLCA incidence and found cigarettes per day, lifetime smoking index and smoking initiation were all associated with increased risk of BLCA 27. Smoking might promote carcinogenesis through activating several cellular signaling pathways. For instance, tobacco components could promote lung cancer cells proliferation and survival through activating AKT and NF-κB signaling pathways 57, 58. Long term exposure to tobacco smoke might induce hepatic cancer stem cell-like properties through IL-33/p38 pathway 59. In addition, cigarette smoke could induce a chronic lung inflammatory microenvironment, oxidative stress and cell structural alterations, which might be attributable to lung tumor growth 60. Repeated exposure to tobacco smoke could trigger IKKβ and JNK1-dependent inflammation and promote lung tumorigenesis 61. Nicotine-derived nitrosamino ketone (NNK) could promote lung cancer formation by upregulating the chemokine CCL20. Dexamethasone, an anti-inflammatory drug, inhibited NNK-induced CCL20 production and suppressed lung cancer *in vitro* and *in vivo* 62. In addition, NNK could induce granulocyte-macrophage colony stimulating factor production and activate CREB to promote pancreatic cancer 63.

Smoking had an obvious effect on blood lipid profiles and could increase dyslipidemia risk 20-24. In Chinese male population, total cholesterol and triglyceride levels were significantly elevated aligned with increasing cigarette smoking 21. Another study based on Chinese population indicated that both the amount and duration of smoking were associated with dyslipidemia risk, and that quitting for more than 6 years reduced dyslipidemia risk 23. For both genders, smoking was associated with higher risk of dyslipidemia, and female smokers might develop dyslipidemia more easily than male smokers 22. Secondhand smoke exposure could increase the risk of dyslipidemia 24, and cigarette smoke was also reported to disrupt pulmonary lipid homeostasis and result in inflammation 64.

Based on above evidences, we regarded the five validated blood lipids as mediators and tried to explore the association between smoking, blood lipids and BLCA. Through mediation MR, we found that both smoking and blood lipids could affect BLCA incidence, and that smoking indirectly affected BLCA through some specific blood lipids.

Our study had several strengths. The primary advantage was the MR approach, which prevented reverse causation and residual confounding. After several methods verifying the fulfilment of MR assumptions, we finally validated that five lipids, including triglycerides in very large HDL, cholesterol in small VLDL, free cholesterol in very large HDL, total free cholesterol, and apolipoprotein B, have a robust and inverse causal association with BLCA. All of them had no heterogeneity or pleiotropy, and presented consistent directions and magnitudes in IVW, weighted median and MR-Egger. The causal inference direction from lipids to BLCA was validated by Steiger test. These results confirmed the robustness of our findings. Secondly, a wide range of exposures including 230 subdivided blood lipids were chosen to perform this MR analysis, which, to the best of our knowledge, is the most comprehensive and systematic study to date to investigate the causal association between lipids and BLCA. Previous studies have focused mainly on total cholesterol, HDL or LDL cholesterol, triglyceride, apolipoprotein, etc., without further classification. In this study, in addition to apolipoprotein B, four other lipids have not been reported before. Our study revealed the causal association between several blood lipids and BLCA, and provided evidence that some subtypes of lipids might play a more important role than others, which indicated that complicated lipids require further classification and reassessment. Thirdly, we used multivariable MR method, which included genetic information on exposures that might be correlated with each other in a joint multivariable model, thus adjusting for other pleiotropic pathways. Moreover, replicative and meta-analysis showed consistent effects and strengthened the causal association between some specific lipids and BLCA. Finally, the association between smoking and BLCA, smoking and lipids, lipids and BLCA have all been separately explored in previous observational studies. However, the systematic analysis of these reported results could be biased by the heterogeneity among different studies, such as differences in selection criteria, population ancestry, and analysis approach. Therefore, we combined smoking, blood lipids and BLCA and elucidated their association by mediation MR in one study.

Several limitations should also be noted. Firstly, the two GWAS studies for blood lipids might have some difference in quality control criteria. The sample size in the GWAS study of Kettunen *et al*. was relatively small 34. Not all the lipids from the primary analysis were detected in the GWAS study of Kettunen *et al*., therefore only blood lipids detected in both studies were chosen for meta-analysis. Secondly, the populations of this study were European, and the results should be validated in other populations before generalizing our findings. Thirdly, other factors, such as gender, diet, medication, age of onset, family history or different histotypes, might affect the MR results 2, 65, 66. However, since the individual level statistics from FinnGen consortium and UKB were not publicly available, we could perform MR analysis only on the basis of summary-level statistics, which lacked the above information for BLCA patients. Future analyses with subdivided groups of BLCA patients could help to clarify the risk factors for BLCA more specifically. Fourthly, in addition to the bladder, the upper urinary tract and proximal urethra were also covered by transitional epithelial cells, and there might be some consistency in the etiology of tumors in these three locations. Since nearly 90% of the urothelial carcinoma was located at bladder, and there were no appropriate GWAS datasets with enough sample sizes for upper tract urothelial carcinoma (UTUC) and urethral cancer, we focused mainly on the risk factors for BLCA in this study. In previous studies, smoking was regarded as the predominant risk factor for BLCA 25 and UTUC 67. The association between smoking and urethral cancer was unclear 68, and the effect of blood lipids on UTUC and urethral cancer has not been previously examined. Thus, further exploration should be conducted to examine the effect of smoking and blood lipids on UTUC and urethral cancer.

In summary, we discovered five lipids to be robustly and inversely associated with BLCA, two of which could attenuate the effect of smoking on BLCA.

## Supplementary Material

Supplementary figures.

Supplementary tables.

## Abbreviations

BLCA: bladder cancer
GWAS: genome-wide association study
HDL: high-density lipoprotein
IVs: instrumental variables
IVW: Inverse variance weighted
LDL: low-density lipoprotein
MR: Mendelian randomization
NNK: Nicotine-derived nitrosamino ketone
RCT: randomized controlled trial
UKB: UK Biobank
UTUC: upper tract urothelial carcinoma
VLDL: very low-density lipoprotein
