# Supplementary material for: Causal association of smoking, blood lipids, and bladder cancer: Insights from a multivariable and mediation mendelian randomization investigation
Source: J Cancer. 2024 Feb 12;15(7):1929–39. doi: 10.7150/jca.92306 (PMC10905390; doi:10.7150/jca.92306)

Supplementary materials

Supplementary Figures S1-S3: Pages 2-24

Supplementary Figures S1-S3

Supplementary Figure S1. Scatter plot results of IVW, weighted median and MR-Egger. Pages 1-11.

Total lipids

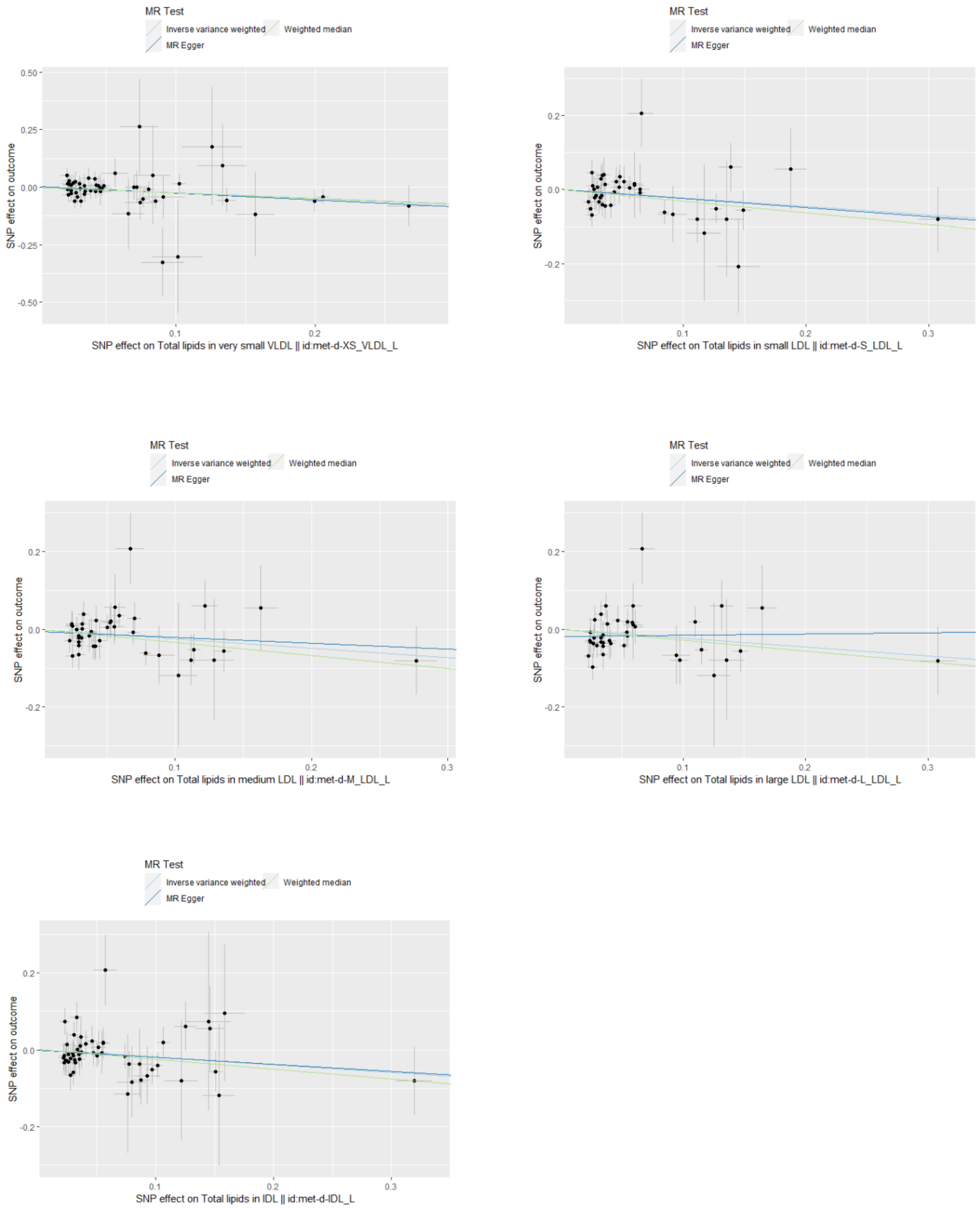

Supplementary Figure S1. Scatter plot results of IVW, weighted median and MR-Egger.

Triglycerides

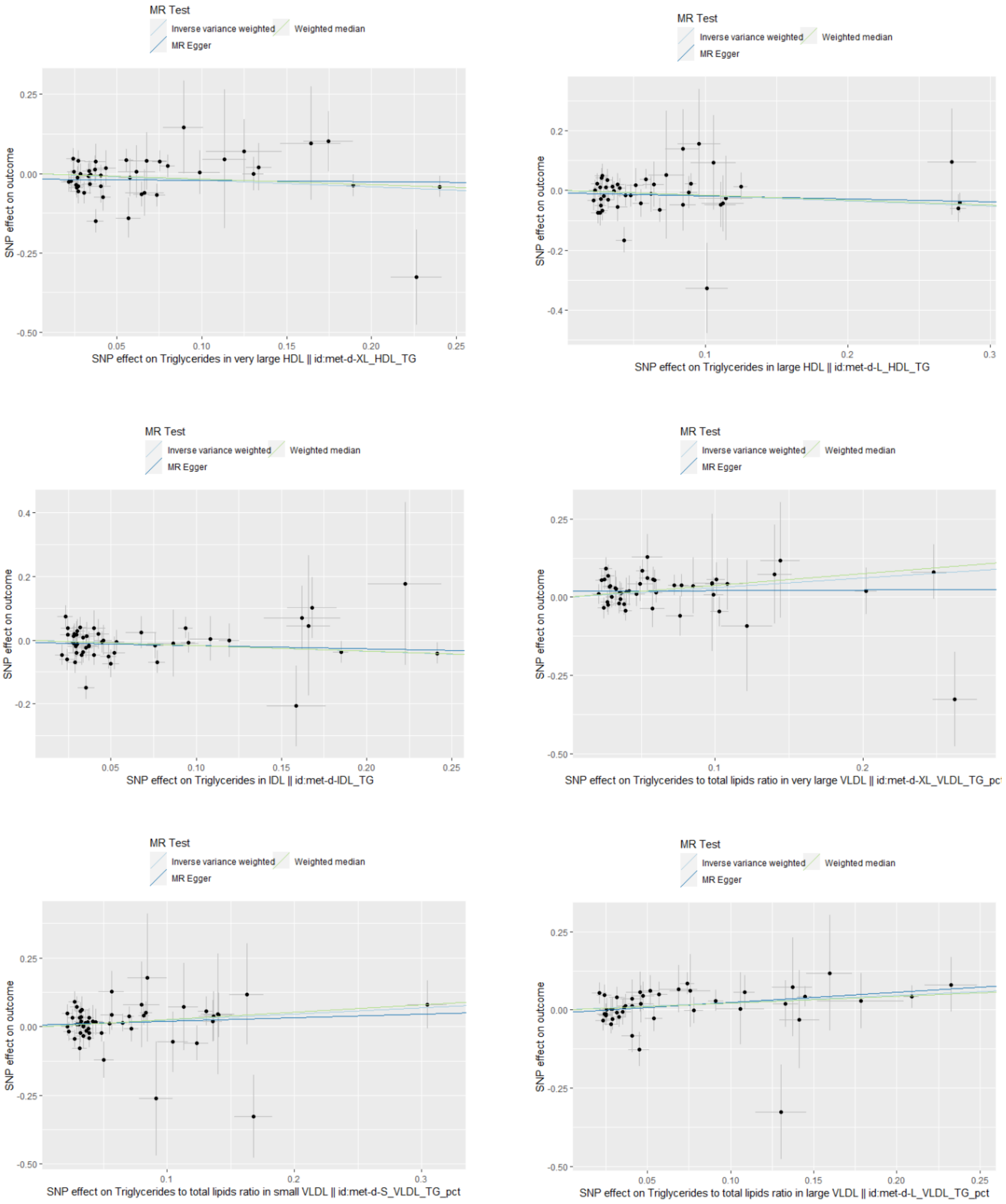

Supplementary Figure S1. Scatter plot results of IVW, weighted median and MR-Egger.

Phospholipids

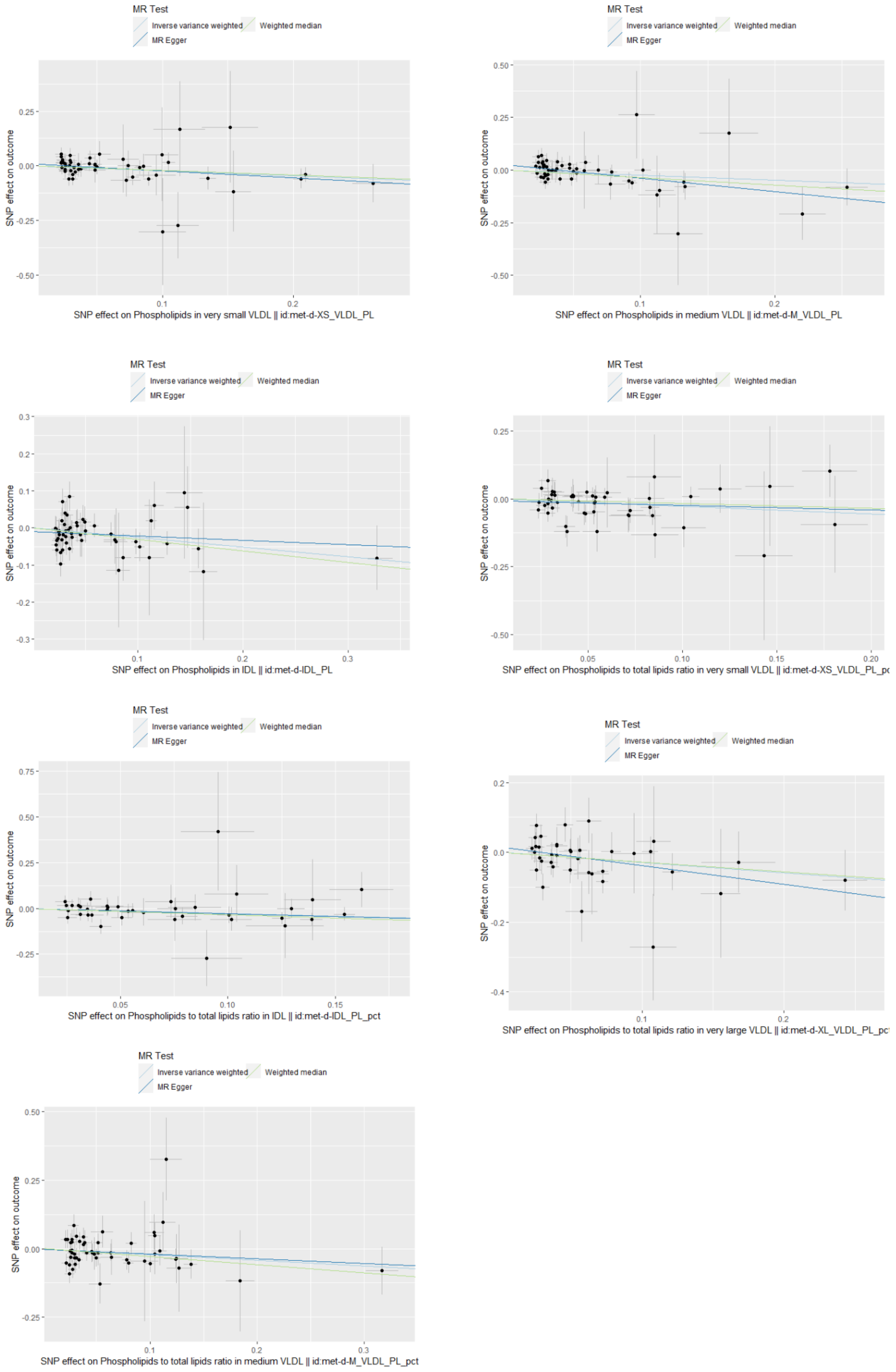

Supplementary Figure S1. Scatter plot results of IVW, weighted median and MR-Egger.

Cholesterol

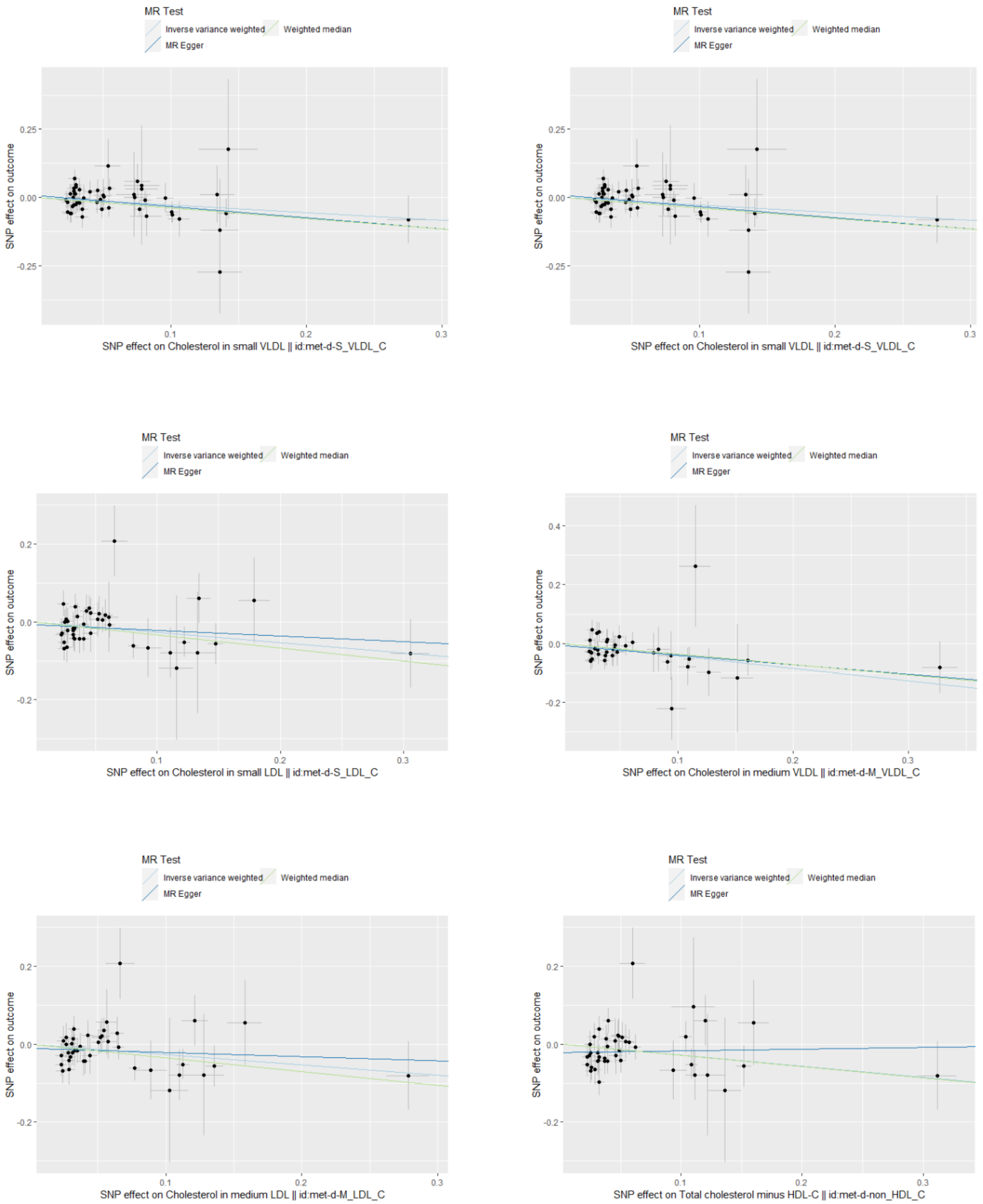

Supplementary Figure S1. Scatter plot results of IVW, weighted median and MR-Egger.

Cholesterol - continued

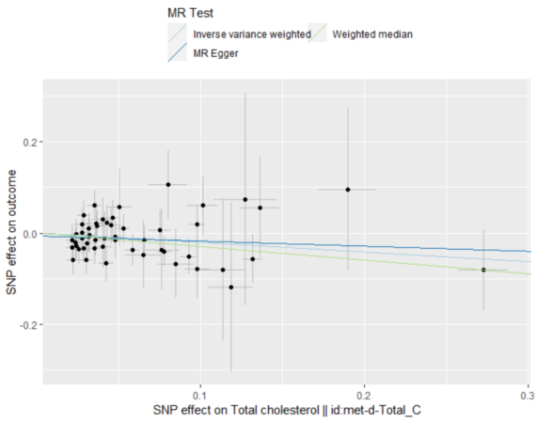

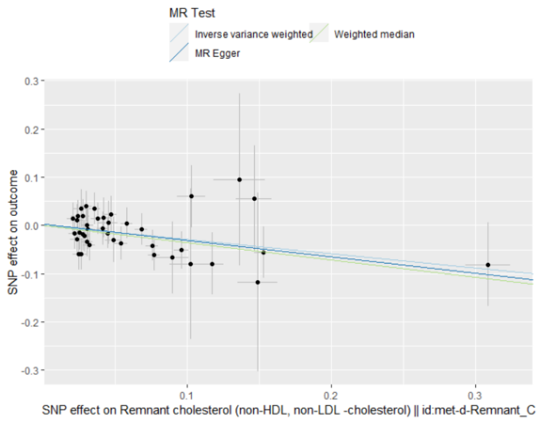

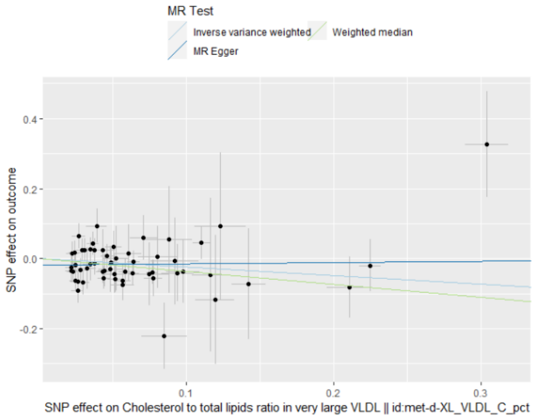

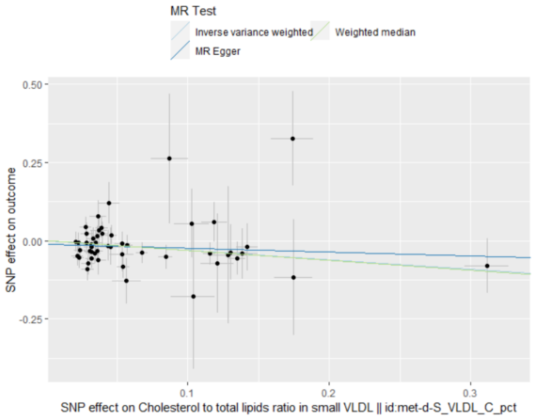

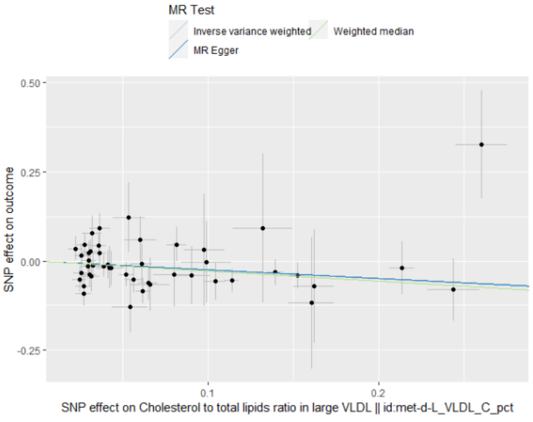

Supplementary Figure S1. Scatter plot results of IVW, weighted median and MR-Egger.

Free cholesterol

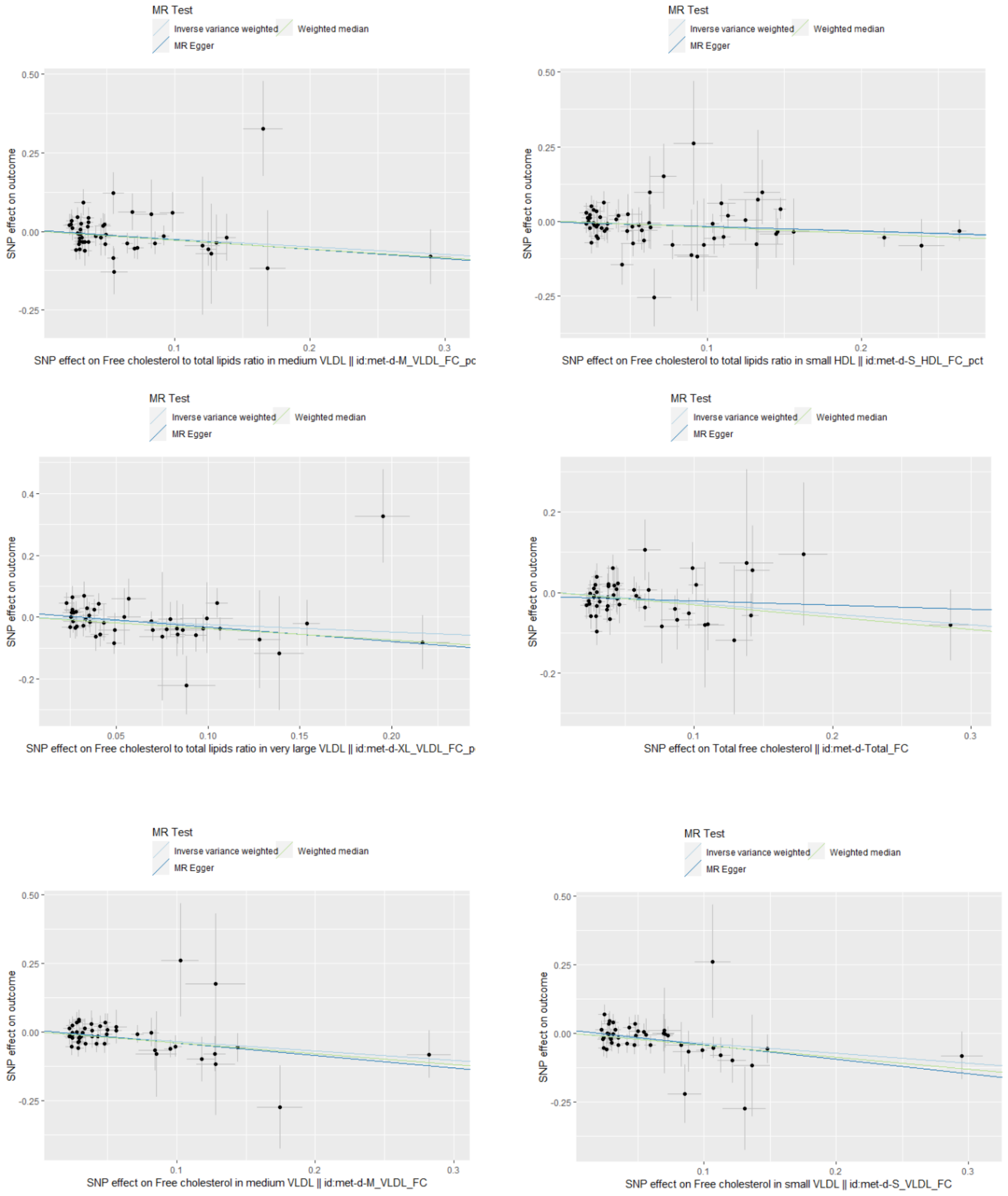

Supplementary Figure S1. Scatter plot results of IVW, weighted median and MR-Egger.

Free cholesterol - continued

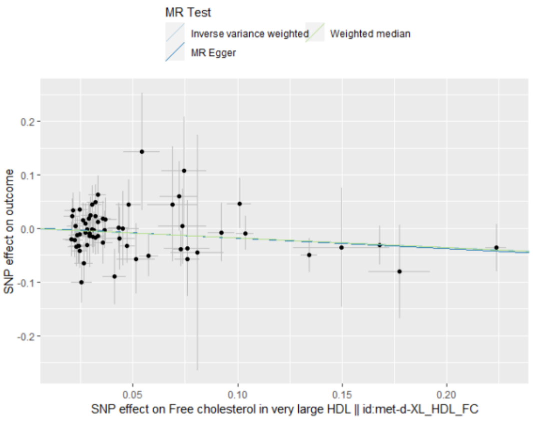

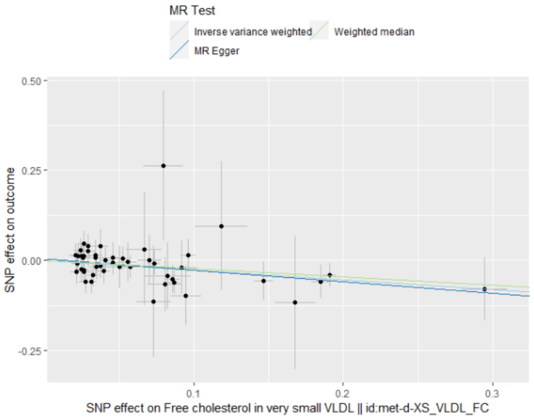

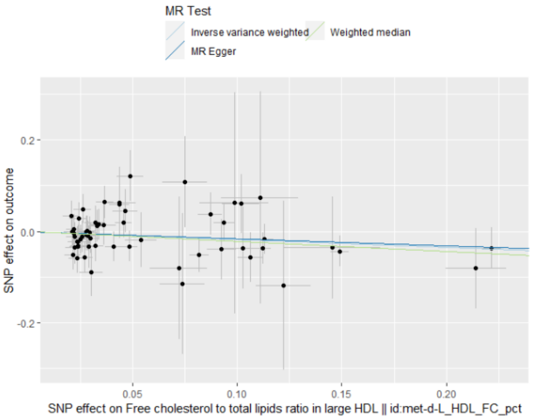

Supplementary Figure S1. Scatter plot results of IVW, weighted median and MR-Egger.

Cholesteryl esters

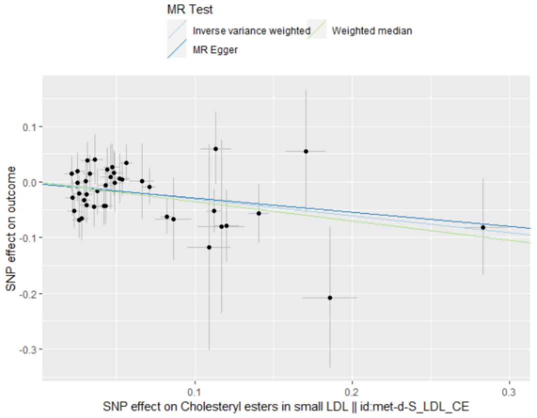

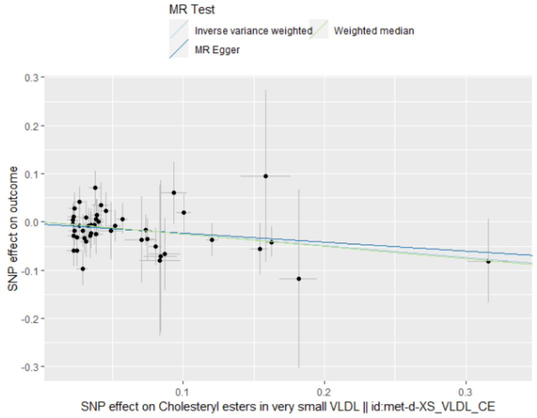

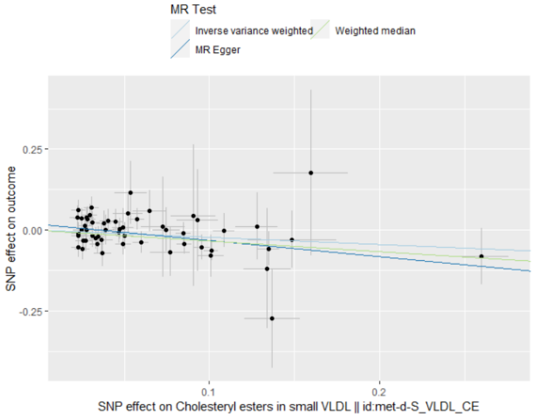

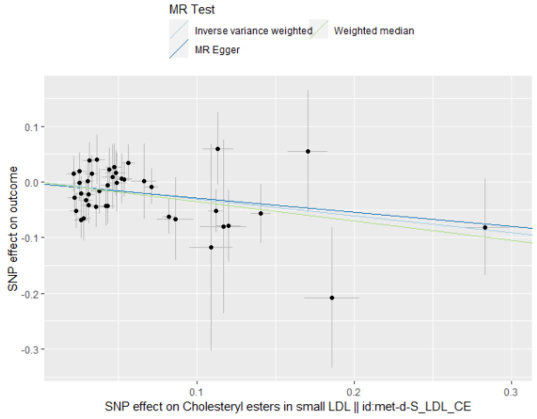

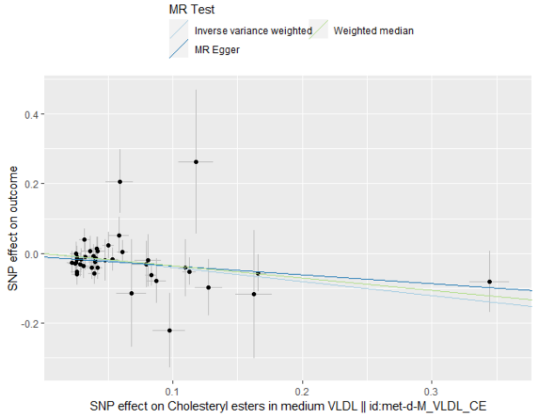

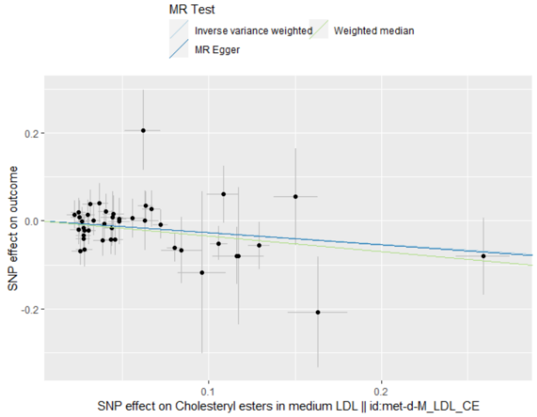

Supplementary Figure S1. Scatter plot results of IVW, weighted median and MR-Egger.

Cholesteryl esters - continued

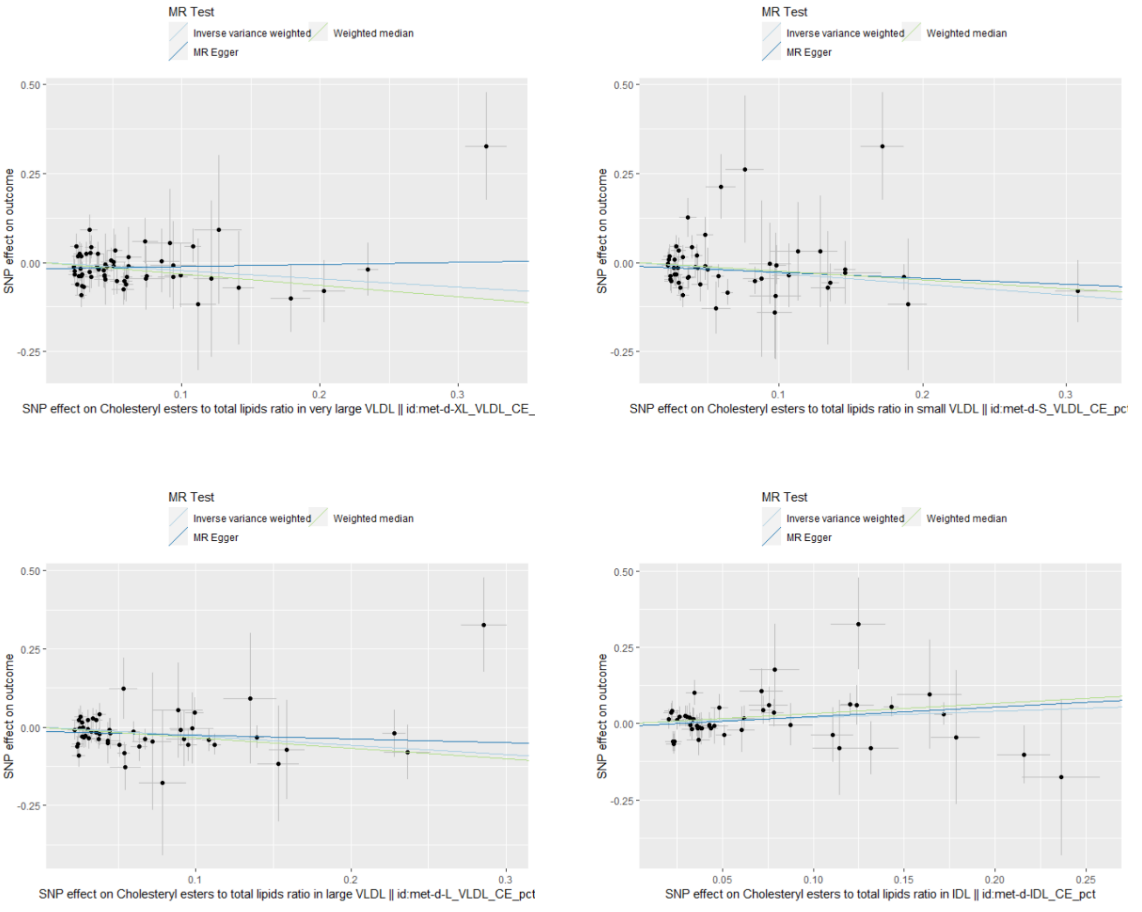

Supplementary Figure S1. Scatter plot results of IVW, weighted median and MR-Egger.

Lipoprotein

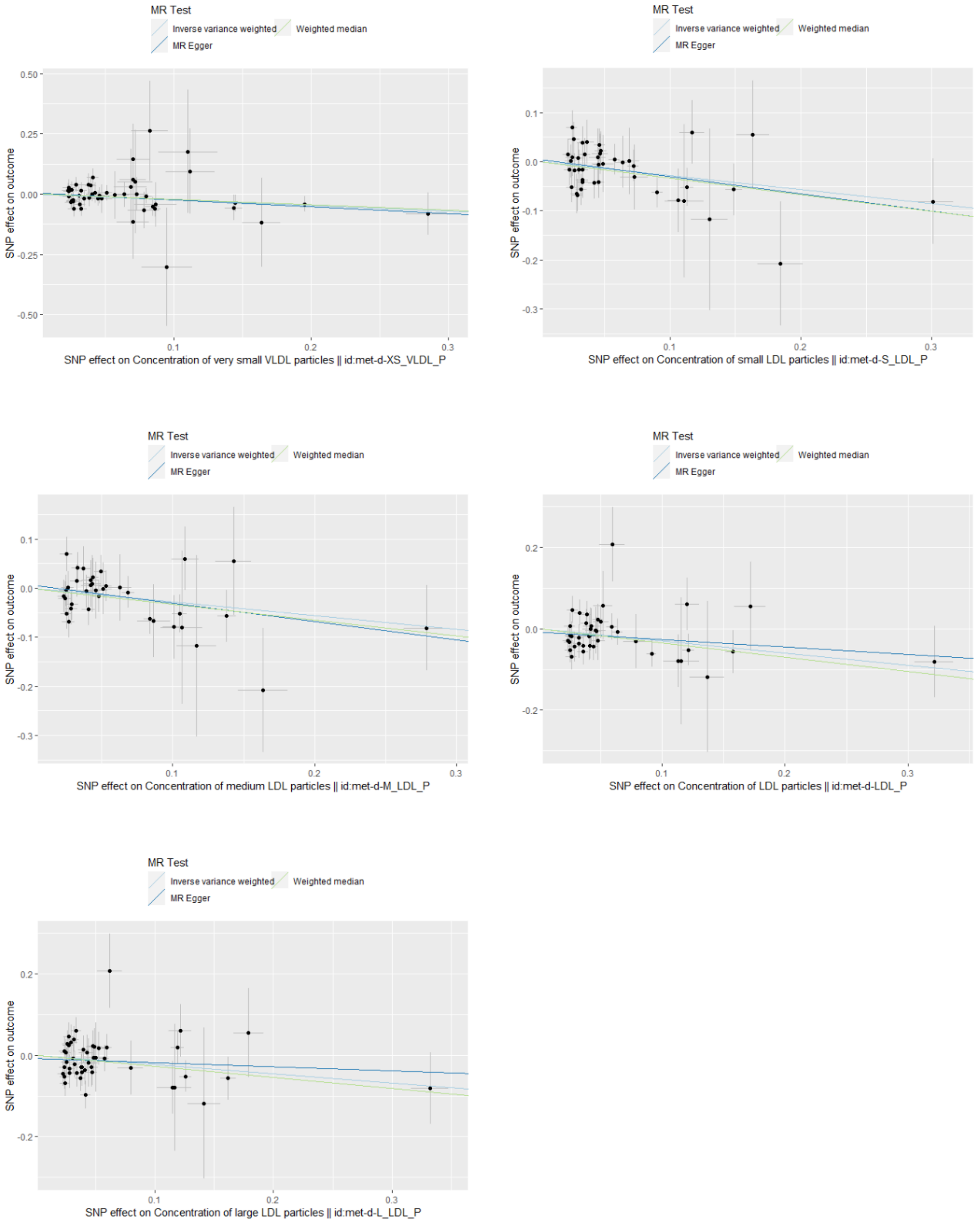

Supplementary Figure S1. Scatter plot results of IVW, weighted median and MR-Egger.

Other

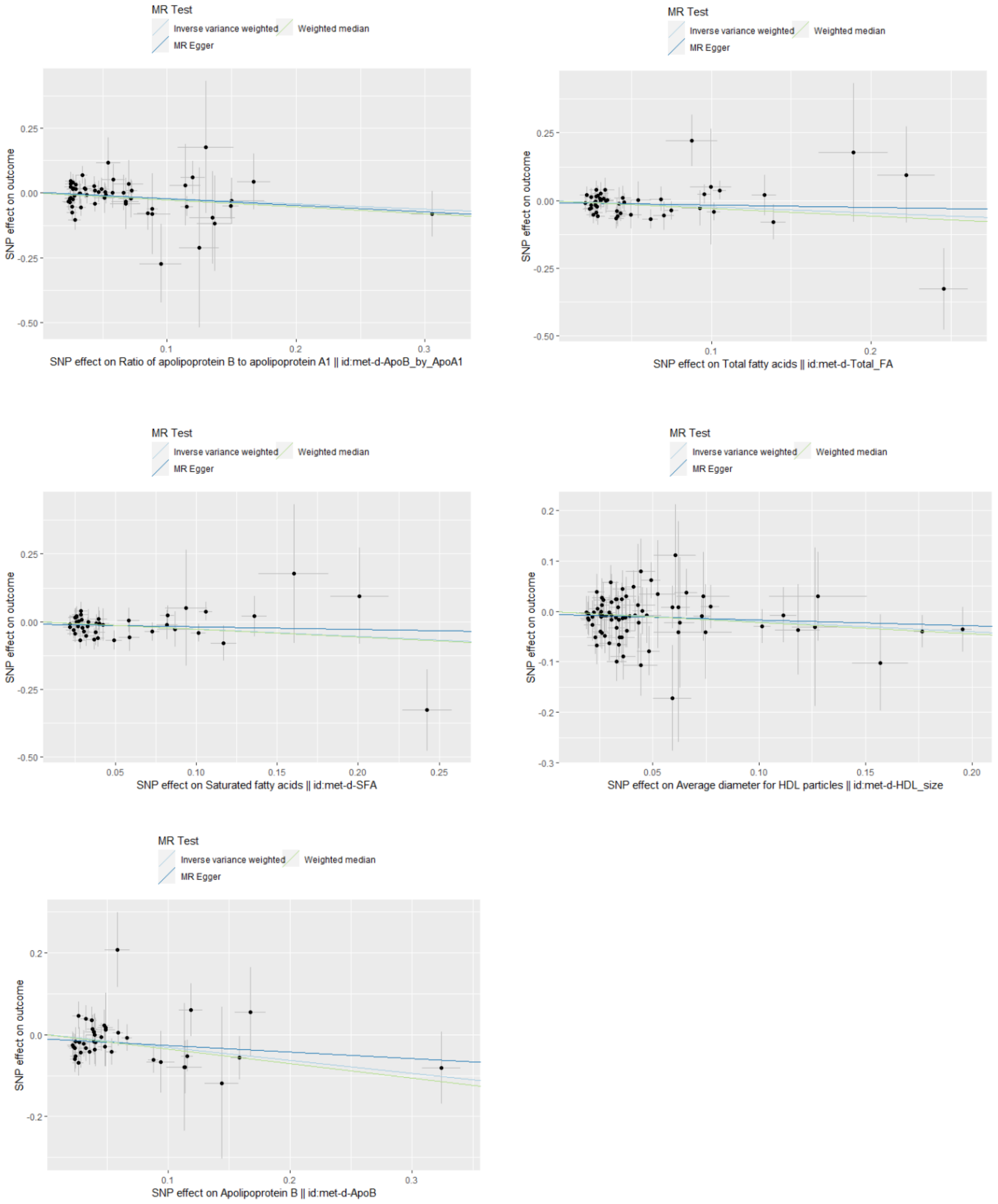

Supplementary Figure S1. Scatter plot results of IVW, weighted median and MR-Egger.

Supplementary Figure S2. Leave-one-out results of IVW, weighted median and MR-Egger. Pages 12-22.

Total lipids

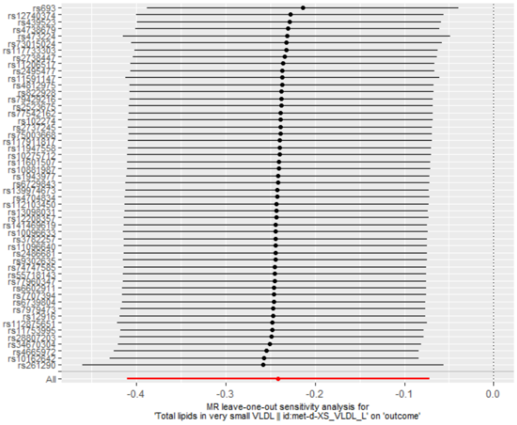

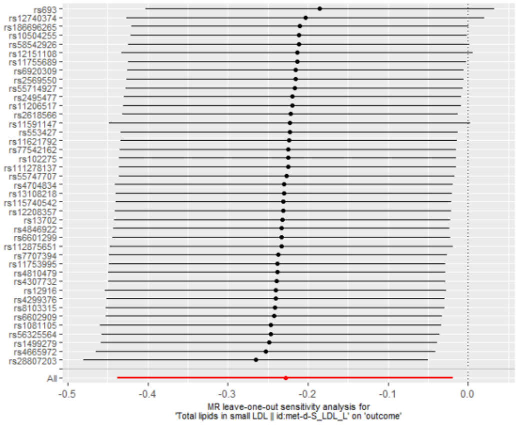

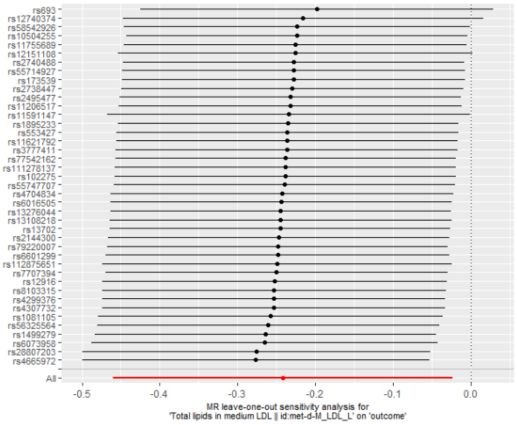

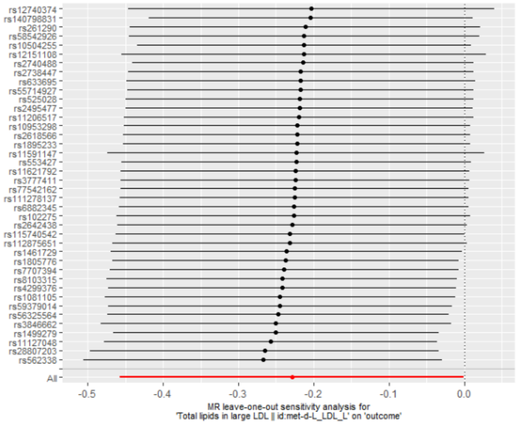

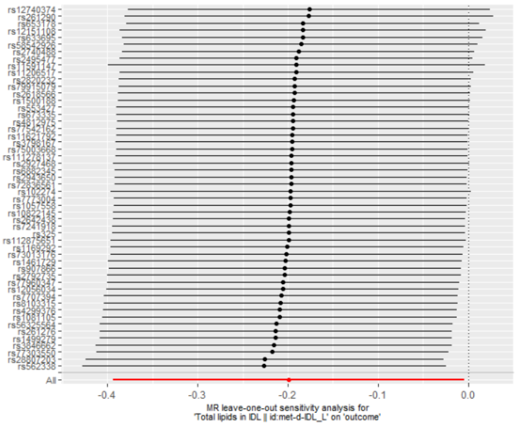

Triglycerides

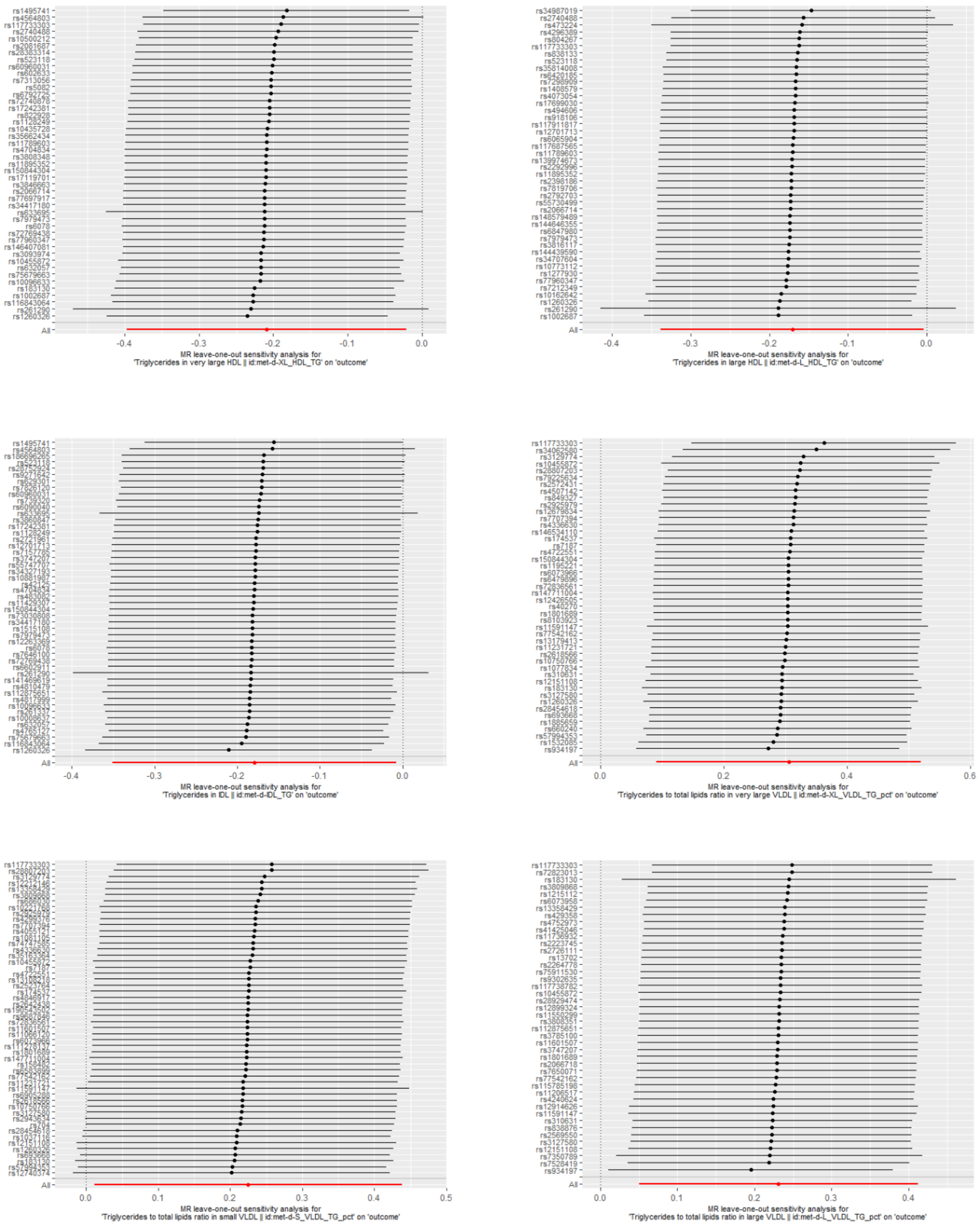

Supplementary Figure S2. Leave-one-out results of IVW, weighted median and MR-Egger.

Phospholipids

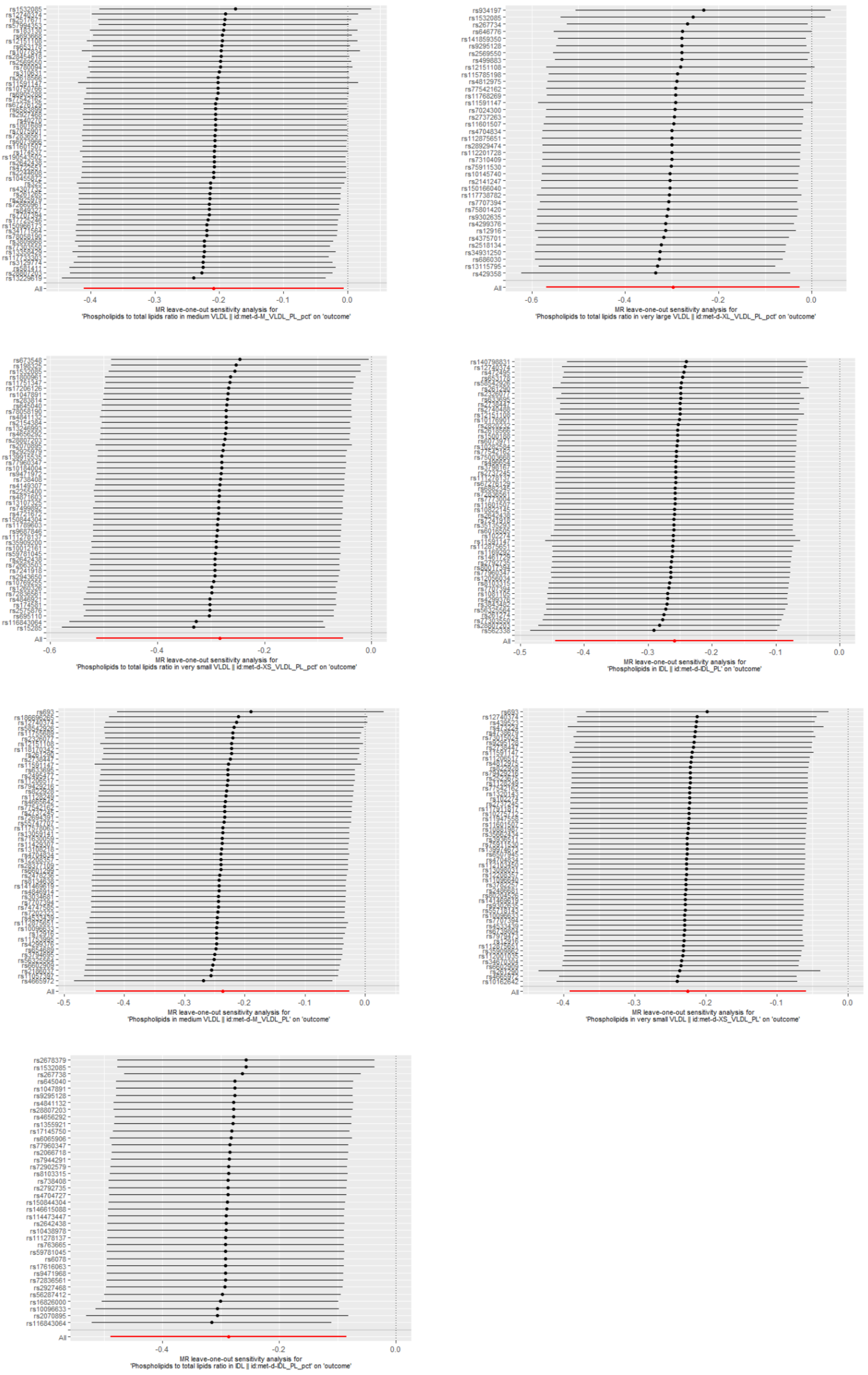

Supplementary Figure S2. Leave-one-out results of IVW, weighted median and MR-Egger.

Cholesterol

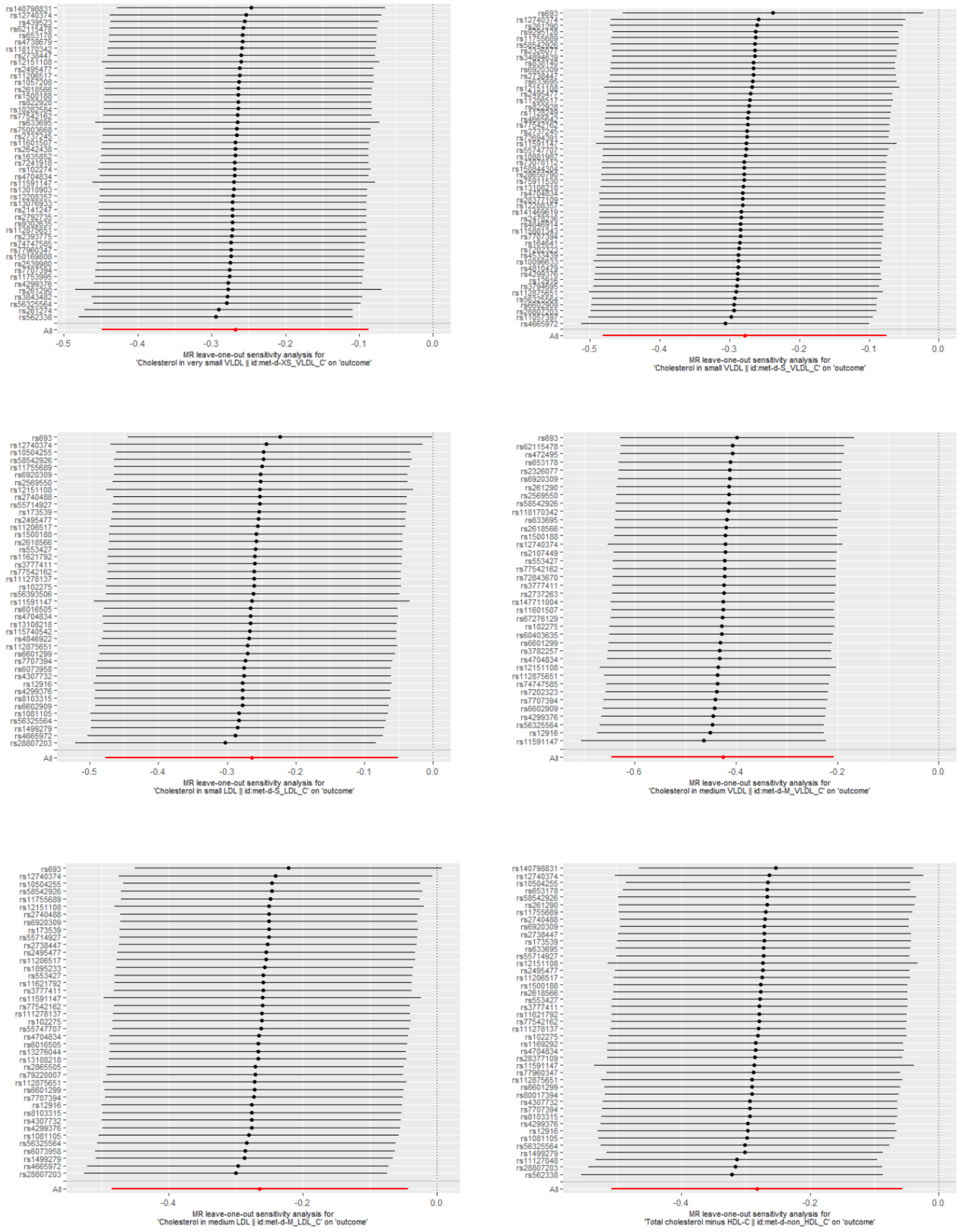

Supplementary Figure S2. Leave-one-out results of IVW, weighted median and MR-Egger.

Cholesterol - continued

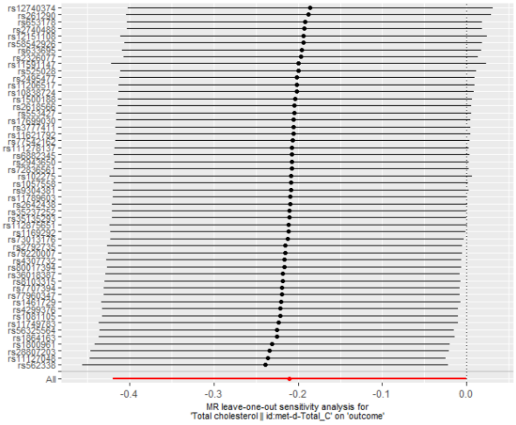

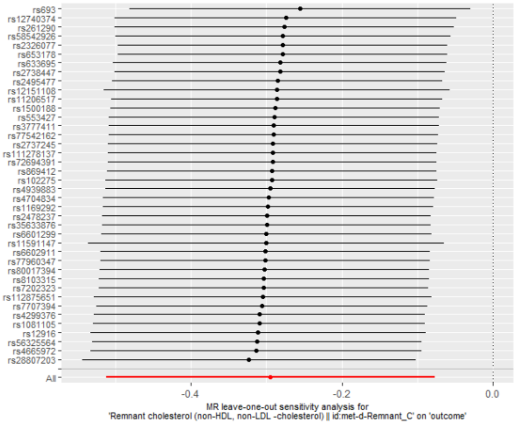

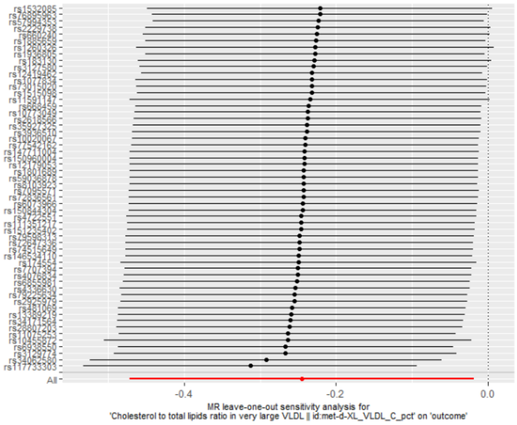

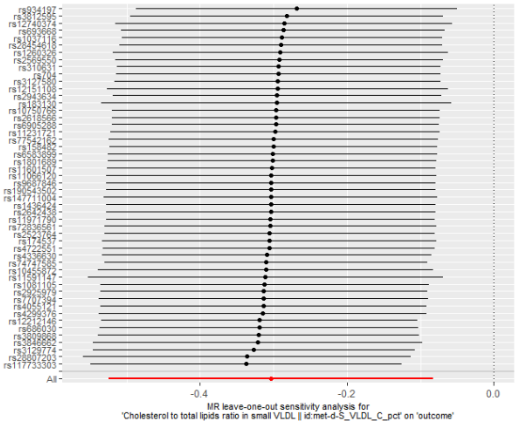

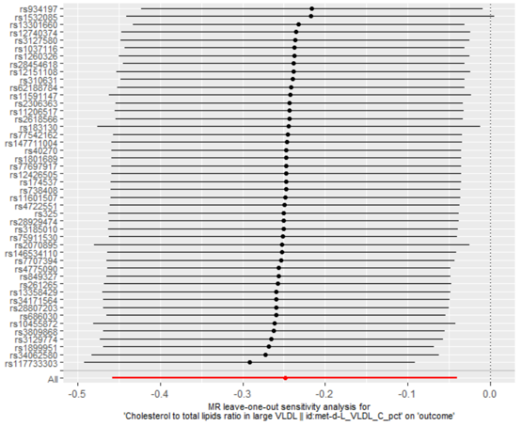

Supplementary Figure S2. Leave-one-out results of IVW, weighted median and MR-Egger.

Free cholesterol

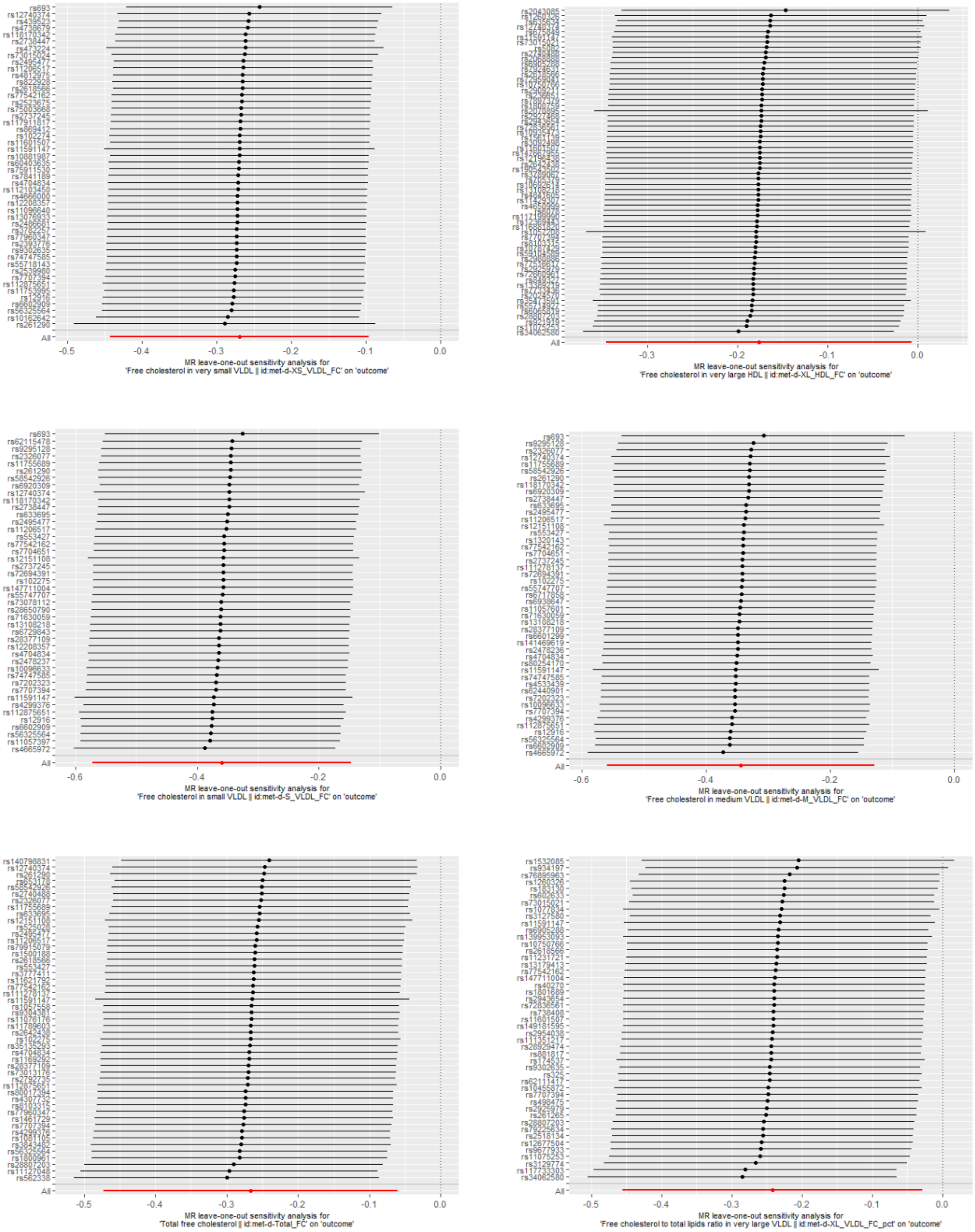

Supplementary Figure S2. Leave-one-out results of IVW, weighted median and MR-Egger.

Free cholesterol - continued

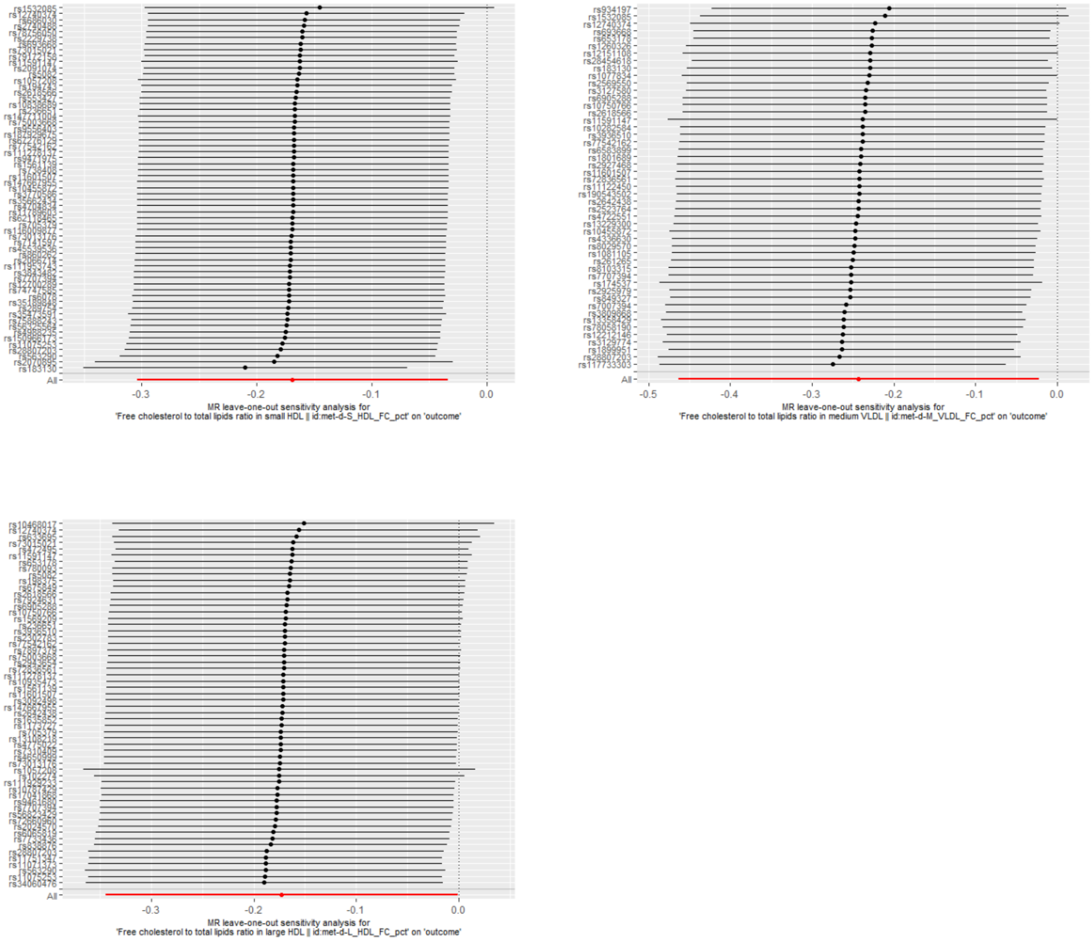

Supplementary Figure S2. Leave-one-out results of IVW, weighted median and MR-Egger.

Cholesteryl esters

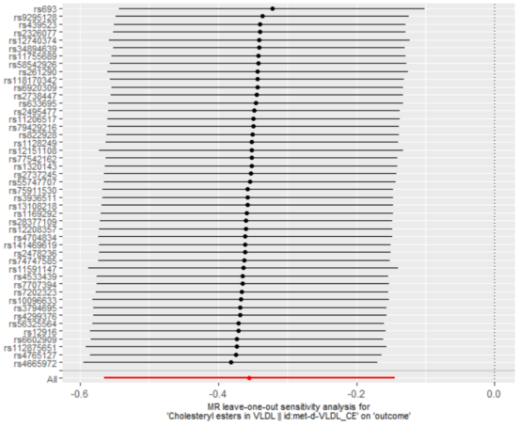

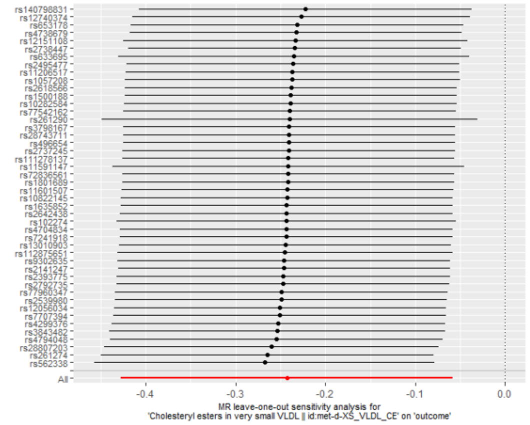

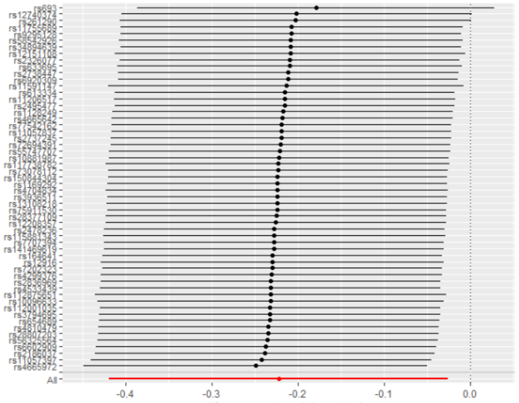

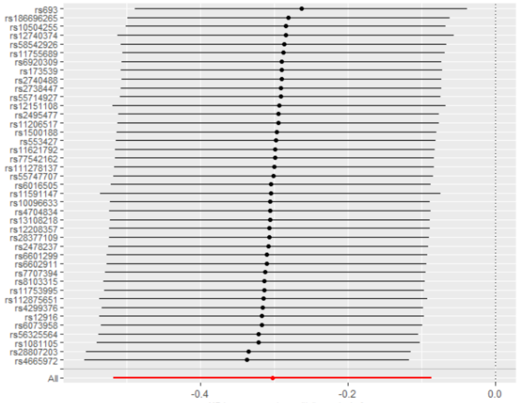

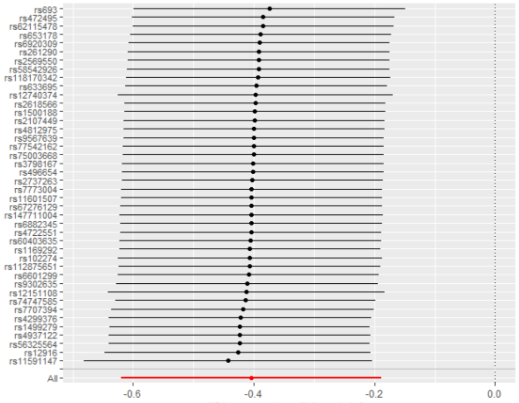

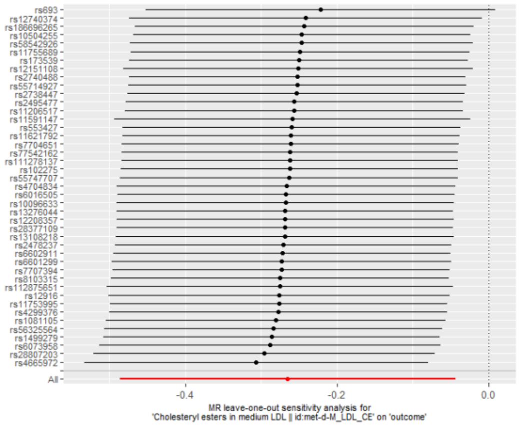

Supplementary Figure S2. Leave-one-out results of IVW, weighted median and MR-Egger.

Cholesteryl esters - continued

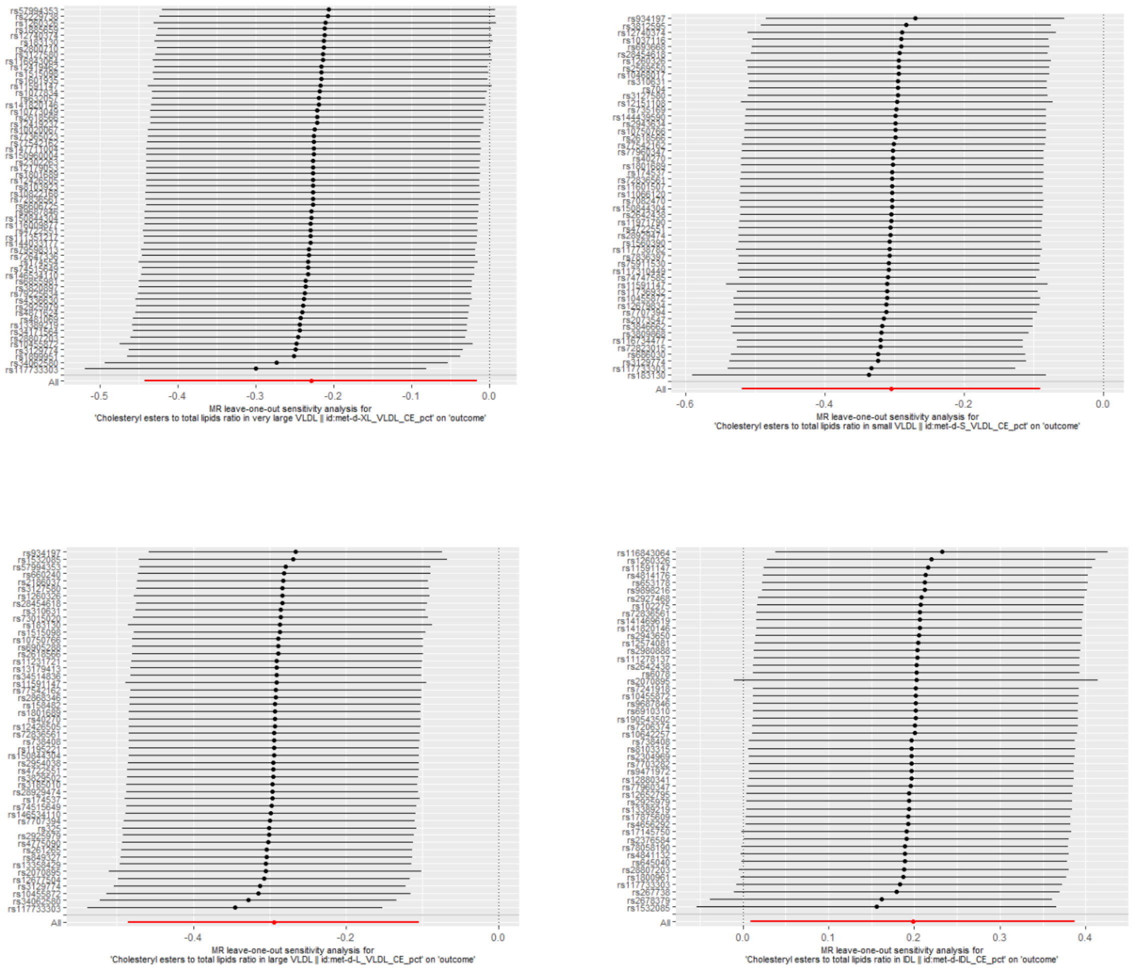

Supplementary Figure S2. Leave-one-out results of IVW, weighted median and MR-Egger.

Lipoprotein

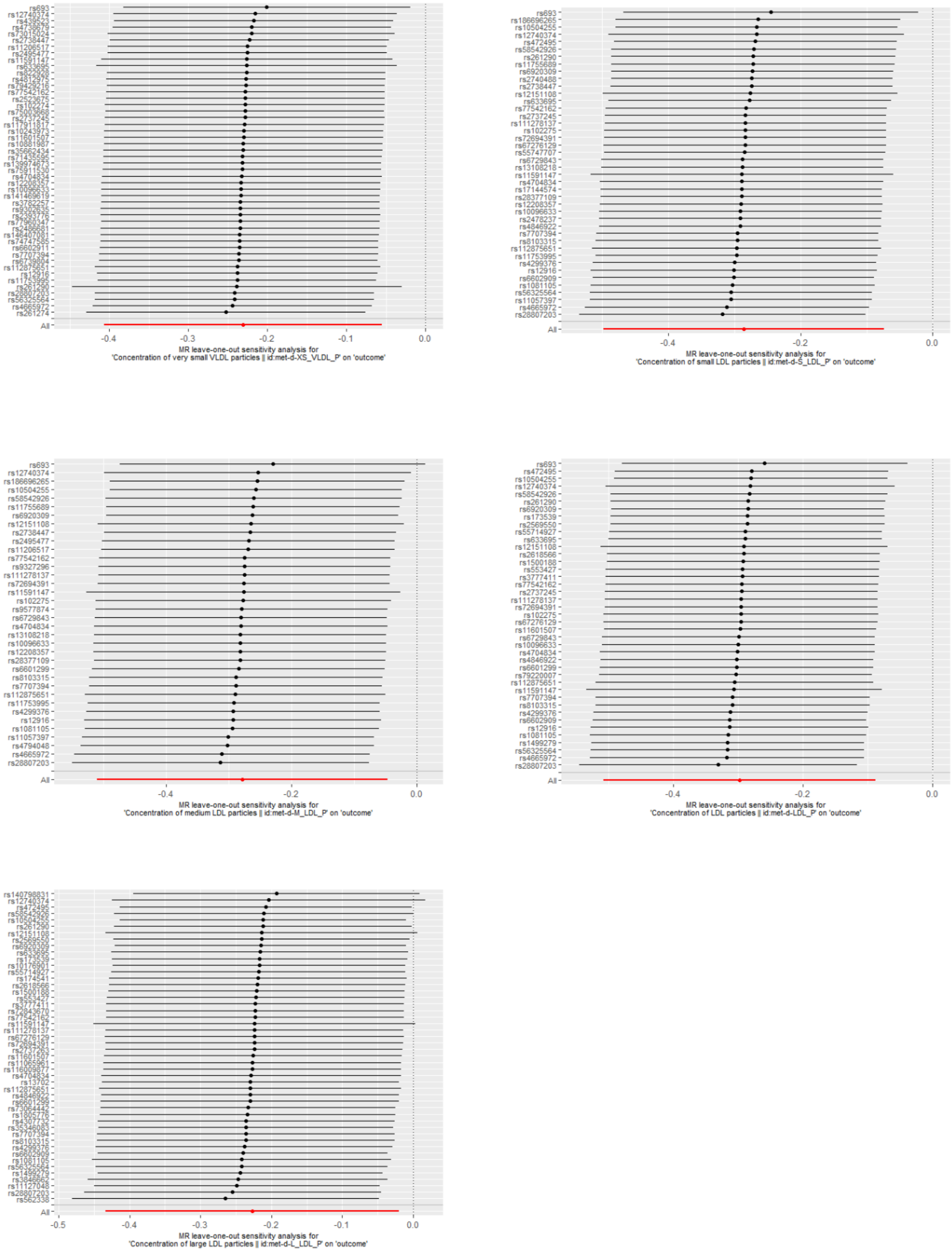

Supplementary Figure S2. Leave-one-out results of IVW, weighted median and MR-Egger.

Other

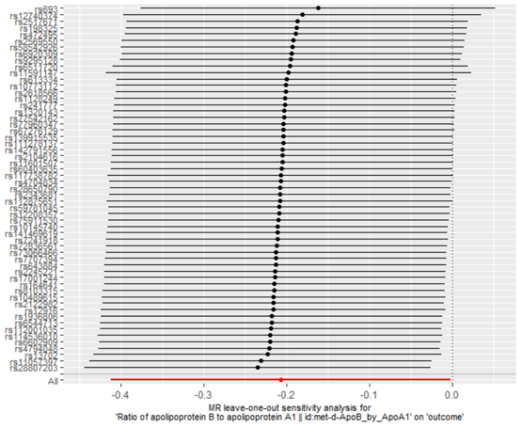

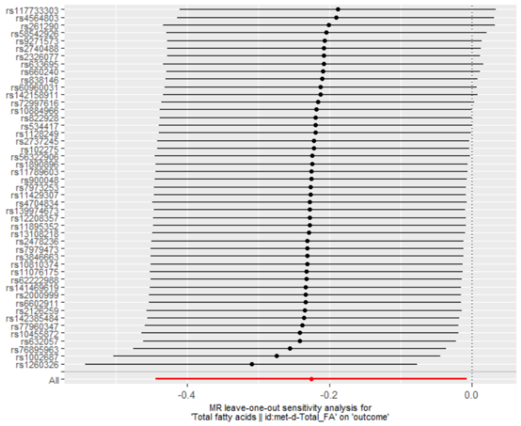

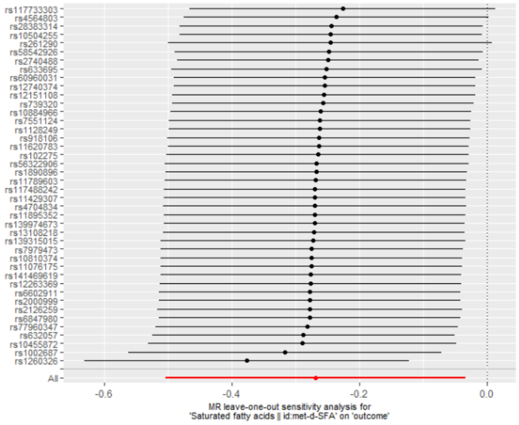

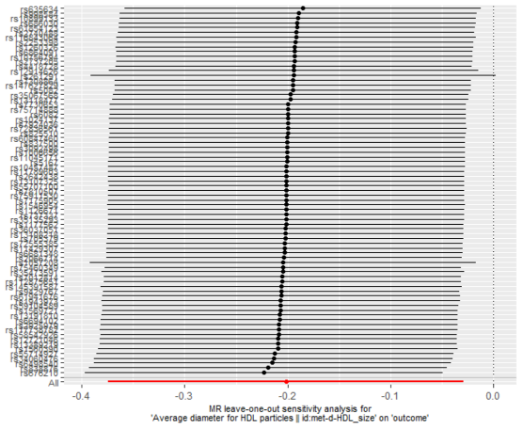

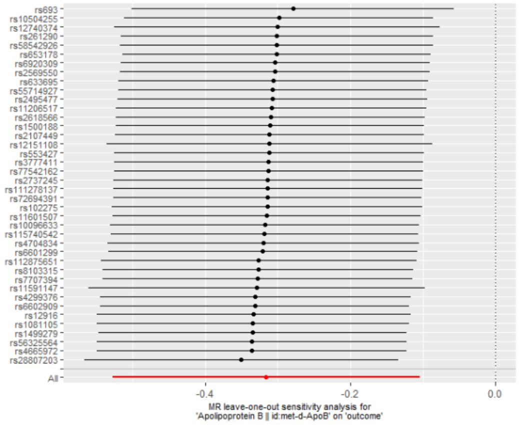

Supplementary Figure S2. Leave-one-out results of IVW, weighted median and MR-Egger.

Supplementary Figure S3. Lipids remained significant after multivariable MR.

Category	SNP	OR(95%CI)	*P*
Total lipids in very small VLDL	31	0.79(0.66-0.95)	0.010
Triglycerides in very large HDL	24	0.84(0.70-1.00)	0.046
Triglycerides in large HDL	29	0.84(0.72-0.99)	0.036
Phospholipids in very small VLDL	31	0.81(0.68-0.96)	0.018
Phospholipids in medium VLDL	30	0.75(0.6-00.95)	0.018
Phospholipids in IDL	30	0.80(0.65-0.99)	0.037
Cholesterol in very small VLDL	28	0.79(0.65-0.96)	0.018
Cholesterol in small VLDL	31	0.74(0.59-0.93)	0.010
Cholesterol in medium VLDL	23	0.68(0.54-0.87)	0.002
Remnant cholesterol (non-HDL, non-LDL -cholesterol)	28	0.74(0.59-0.94)	0.012
Cholesterol to total lipids ratio in small VLDL	27	0.75(0.58-0.98)	0.034
Free cholesterol in very small VLDL	28	0.75(0.63-0.91)	0.003
Free cholesterol in small VLDL	27	0.70(0.56-0.89)	0.003
Free cholesterol in medium VLDL	26	0.74(0.58-0.94)	0.013
Total free cholesterol	29	0.76(0.60-0.96)	0.019
Cholesteryl esters in VLDL	29	0.71(0.57-0.90)	0.004
Cholesteryl esters in very small VLDL	27	0.81(0.67-1.00)	0.045
Cholesteryl esters in small VLDL	33	0.76(0.61-0.95)	0.015
Cholesteryl esters in medium VLDL	23	0.72(0.56-0.92)	0.008
Cholesteryl esters to total lipids ratio in IDL	28	1.26(1.00-1.58)	0.046
Concentration of very small VLDL particles	30	0.80(0.66-0.96)	0.017
Concentration of medium LDL particles	22	0.77(0.60-1.00)	0.049
Total fatty acids	25	0.75(0.60-0.95)	0.017

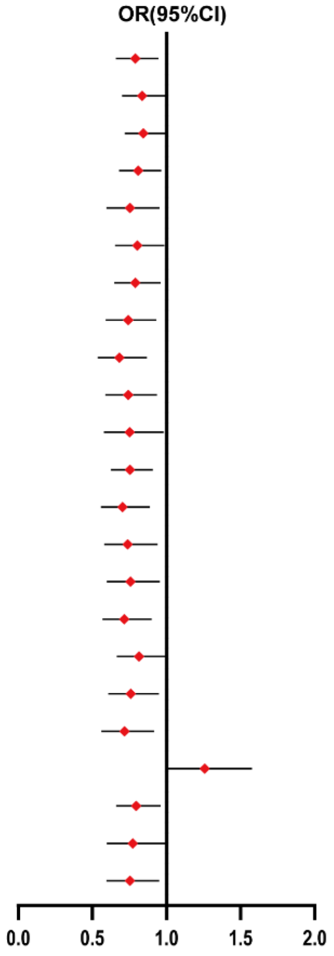

Supplement: Supplementary file 1 — Supplementary figures. [file jcav15p1929s1.pdf]
